# Phosphoric Acid-Modified Wood Biochar for Cr(VI) Retention in Aqueous Systems and Sustained Mobility Control Under Dynamic Soil Conditions

**DOI:** 10.3390/molecules31203608

**Published:** 2026-10-10

**Authors:** Zihan Su, Sitong Li, Junfeng Tang, Zipeng Tu, Lipin Ren, Bin He, Zihao Duan, Wenshuang Yu

**Affiliations:** 1 College of Civil Engineering, Sichuan Agricultural University, Dujiangyan 611830, China; suzihan200603@163.com (Z.S.); 19981517227@163.com (S.L.); 13060028978@163.com (Z.T.); 13980240859@163.com (L.R.); 15928818225@163.com (B.H.); wsdzhdsg@163.com (Z.D.); 19123392582@163.com (W.Y.); 2 Sichuan Higher Education Engineering Research Center for Disaster Prevention and Mitigation of Village Construction, Sichuan Agricultural University, Dujiangyan 611830, China

**Keywords:** phosphoric acid engineering, wood biochar amendment, Cr(VI) retention, soil migration control, repeated infiltration, drying–rehumidification

## Abstract

Effective remediation of hexavalent chromium [Cr(VI)] requires materials that perform well not only in aqueous systems but also in heterogeneous soils exposed to fluctuating water flow and environmental disturbance. Here, phosphoric acid–modified wood biochar (P-BC) was evaluated against pristine biochar (BC) through physicochemical characterization, pH-dependent aqueous retention tests, soil water-retention measurements, repeated-infiltration leaching experiments, and accelerated drying–rehumidification cycles. Across the tested pH range (pH 2.0–4.0), P-BC showed slightly higher mean apparent Cr(VI) retention than BC at each tested pH. Batch experiments showed higher measured apparent Cr(VI)-derived retention for P-BC under the tested conditions: at 25 °C and an initial Cr(VI) concentration of 106.06 mg L^−1^, the 360-min mean apparent retention values were 8.46 ± 0.03 mg g^−1^ for BC and 10.10 ± 0.10 mg g^−1^ for P-BC. At 55 °C, the model-derived Langmuir q_max_ values were 18.08 and 28.18 mg g^−1^ for BC and P-BC, respectively. P-BC showed higher late-stage water-retention values, while a 3.5% layered application reduced cumulative Cr(VI) leaching by 82.9% relative to the unamended control. Under the accelerated drying–rehumidification protocol, low mean water-extractable Cr(VI) endpoints were observed at the higher P-BC application rates, with the 2.5% P-BC treatment yielding the lowest mean cycle-12 concentration among the tested P-BC treatments. Taken together, these findings show that phosphoric acid modification was associated with modestly higher aqueous Cr(VI)-derived retention and lower measured Cr(VI) mobility under several dynamic soil conditions. The results further indicate that amendment rate and spatial configuration are important determinants of remediation performance, supporting further evaluation of P-BC as an engineered amendment for aqueous Cr(VI) treatment and soil mobility control.

## 1. Introduction

Chromium contamination remains a persistent environmental concern because Cr can migrate through connected soil–water systems and accumulate along exposure pathways. Among its environmentally relevant oxidation states, hexavalent chromium [Cr(VI)] warrants particular attention: its oxyanionic species are highly mobile in aqueous environments and considerably more toxic than trivalent chromium [Cr(III)]. Industrial activities such as electroplating, leather processing, metallurgy, mining, and pigment production can introduce Cr into soils and groundwater, creating persistent risks of contaminant transport and human exposure [1,2]. Conventional remediation methods, including precipitation, ion exchange, membrane separation, electrochemical treatment, and stabilization, can perform effectively under controlled conditions. Their field application in heterogeneous soils, however, is often constrained by high costs, secondary waste generation, and inconsistent performance [2,3]. These limitations underscore the need for affordable amendments that retain Cr(VI) while remaining effective under fluctuating water flow and environmental disturbance.

Biochar is well suited to Cr(VI) remediation because its surface functional groups, mineral phases, and redox-active domains may jointly contribute to Cr(VI) retention through electrostatic attraction, surface complexation, interfacial retention, and redox transformation [2]. Even so, pristine biochar commonly shows limited affinity for anionic Cr(VI), and its performance varies with feedstock properties, pyrolysis conditions, solution pH, surface charge, and surface chemistry [4,5,6]. Phosphoric acid modification offers one route to address these limitations by altering surface morphology and introducing oxygen- and phosphorus-containing functionalities. Previous studies have reported enhanced Cr(VI) removal by phosphoric acid–modified biochars compared with their parent materials. In addition, C–P–O domains have been identified as reactive interfacial sites associated with Cr(VI) adsorption and partial reduction [7]. However, demonstrating improved Cr(VI) removal in a well-mixed aqueous system does not establish that the same modification will provide sustained contaminant control in soil, where water flow, amendment placement, pore connectivity, and environmental disturbance become additional controlling factors. Accordingly, the unresolved question is not simply whether phosphoric acid modification increases Cr(VI) retention, but whether this material-scale improvement remains functionally relevant under dynamic soil conditions.

Most evidence concerning modified biochars comes from well-mixed aqueous systems, whereas soil performance also depends on transient water flow, pore connectivity, amendment placement, and environmental disturbance [6,8,9,10,11]. Biochar-induced changes in soil hydrology can alter pore-water residence time and solute transport, while localized reactive zones may intercept advective Cr(VI) migration [3,12]. A key question is therefore whether enhanced aqueous reactivity translates into sustained Cr(VI) mobility control under dynamic soil conditions [13,14,15]. Our recent study examined H_2_O_2_-functionalized woody biochar using a related application-oriented framework, with emphasis on oxidative surface functionalization and adsorption-coupled Cr(VI) transformation [16]. The present study addresses a different modification pathway by examining how H_3_PO_4_-induced phosphorus/oxygen-containing surface chemistry and textural restructuring, together with amendment configuration, influence Cr(VI) retention and mobility control under dynamic soil conditions. Because the pristine BC materials in the two studies were independently prepared under different pyrolysis conditions, cross-study comparisons are restricted to the level of modification strategy and resulting material behavior rather than direct comparisons of absolute physicochemical properties or Cr(VI)-retention performance. We hypothesized that phosphoric acid modification would alter the pore structure, interfacial chemistry, and surface-charge properties of wood-derived biochar, thereby enhancing apparent aqueous Cr(VI)-derived retention and strengthening Cr(VI) mobility control under dynamic soil conditions.

To address this gap, pristine wood-derived biochar (BC) was modified with phosphoric acid to obtain phosphoric acid–modified biochar (P-BC), and both materials were evaluated in complementary aqueous and soil systems. The study first characterized modification-induced changes in surface morphology, textural properties, surface chemistry, elemental composition, and surface-charge properties using scanning electron microscopy coupled with energy-dispersive X-ray spectroscopy (SEM–EDS), N_2_ adsorption–desorption analysis, Fourier transform infrared spectroscopy (FTIR), X-ray photoelectron spectroscopy (XPS), the point of zero charge (pH_pzc_), and zeta-potential measurements. Aqueous Cr(VI) retention was then examined under different acidic pH values, contact times, initial concentrations, and temperatures, with kinetic and isotherm models used as empirical descriptors rather than definitive mechanistic evidence. The soil experiments assessed the influence of P-BC on water-retention behavior, compared homogeneous and layered amendment configurations during repeated infiltration at different application rates, and evaluated the persistence of Cr(VI) attenuation during successive drying–rehumidification cycles. Taken together, this framework places soil-scale transport control and environmental stability at the center of the performance assessment, while the aqueous experiments provide complementary evidence of material-scale reactivity.

## 2. Results and Discussion

### 2.1. Physicochemical Characteristics of BC and P-BC

#### 2.1.1. Surface Morphology

**Figure 1:**
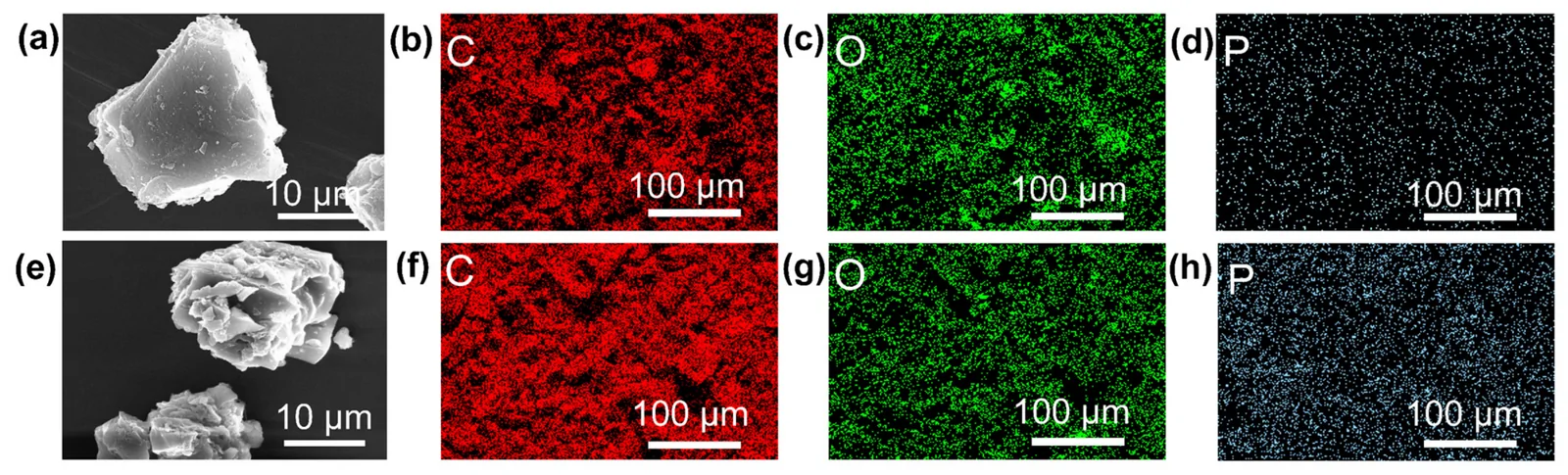
SEM images of pristine BC (**a**) and P-BC (**e**), with corresponding EDS elemental maps of C, O, and P for BC (**b**–**d**) and P-BC (**f**–**h**), respectively. Scale bars: 10 μm for SEM images and 100 μm for EDS maps.

SEM observations revealed clear morphological differences between pristine BC and P-BC (Figure 1a,e). BC exhibited a relatively compact and smooth surface, with limited fissures and exposed pore openings. After phosphoric acid modification, P-BC developed a markedly rougher and more heterogeneous morphology. The particles appeared more fragmented and wrinkled, accompanied by layered structures, surface depressions, fissures, and irregular voids. These observations indicate that phosphoric acid treatment substantially reorganized the external morphology of the wood-derived biochar and increased the exposure of visible surface features.

The more heterogeneous and fissured morphology of P-BC indicates clear modification-induced restructuring of the external surface. Because SEM provides primarily qualitative morphological information, however, these observations alone cannot establish changes in specific surface area, total pore volume, or pore-size distribution. These textural properties were therefore characterized by N_2_ adsorption–desorption analysis, as described in Section 2.1.2.

#### 2.1.2. Textural Properties and Pore Structure

**Figure 2:**
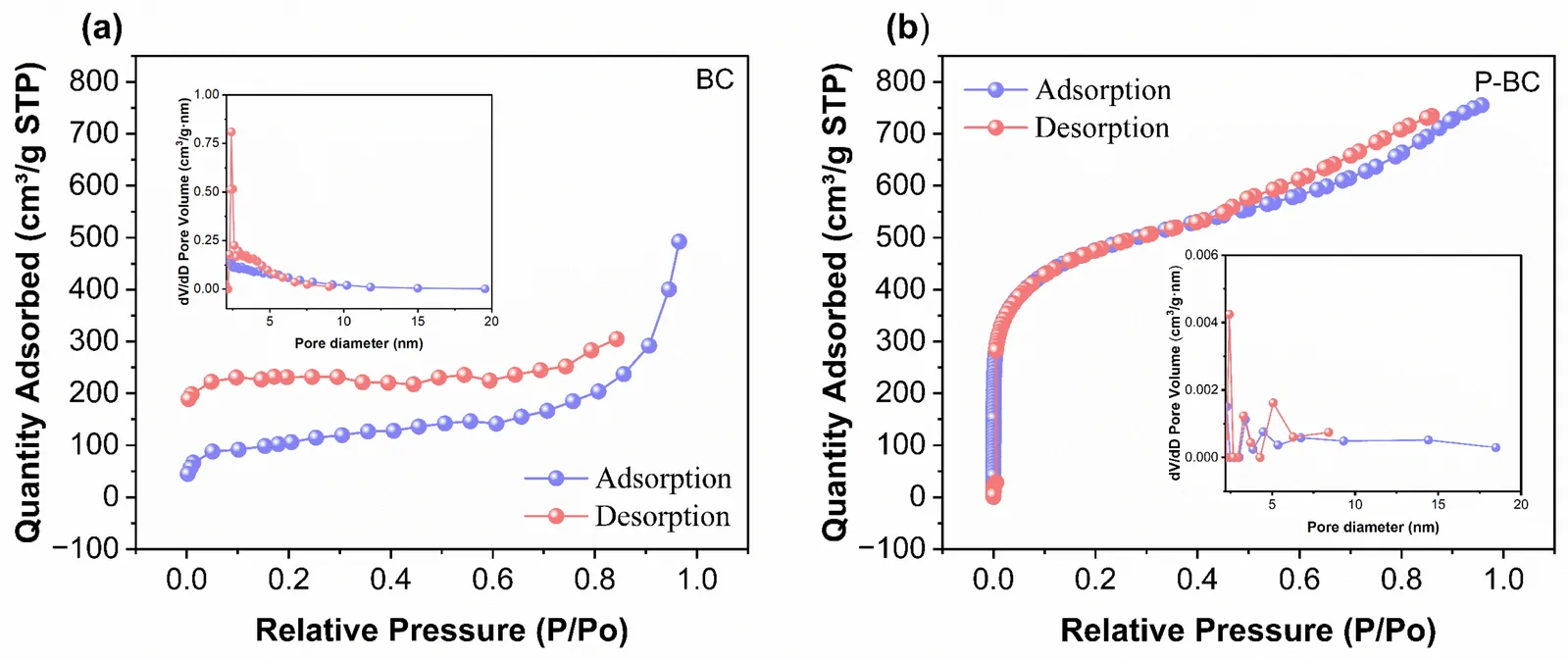
N_2_ adsorption–desorption isotherms of (**a**) pristine biochar (BC) and (**b**) phosphoric acid–modified biochar (P-BC) at 77.35 K, plotted using identical axis scales. Insets show the corresponding BJH mesopore-size distributions; these profiles are not used to characterize micropores.

N_2_ adsorption–desorption measurements indicated that phosphoric acid modification increased the estimated BET specific surface area from approximately 280 m^2^ g^−1^ for BC to approximately 500 m^2^ g^−1^ for P-BC, corresponding to an increase of about 80%. The total pore volume increased from 0.2006 to 0.3398 cm^3^ g^−1^, while the average pore diameter increased from 2.7 to 4.1 nm. The adsorption–desorption profiles also changed markedly after phosphoric acid modification (Figure 2a,b). Relative to BC, P-BC exhibited a distinctly different isotherm profile together with a larger N_2_-accessible surface area and total pore volume, consistent with substantial restructuring of the accessible pore network. The separation between the adsorption and desorption branches, particularly for BC, indicates hysteretic adsorption–desorption behavior within a heterogeneous pore system. Because the present N_2_-sorption data do not uniquely resolve pore geometry or connectivity, this branch separation is interpreted descriptively rather than assigned to a specific pore architecture. Taken together, the N_2_-sorption results provide comparative evidence of modification-induced changes in pore accessibility and textural structure, but are not considered direct evidence of a specific Cr(VI)-retention or mass-transfer mechanism.

#### 2.1.3. Surface Functional Groups and Phosphorus Incorporation

**Figure 3:**
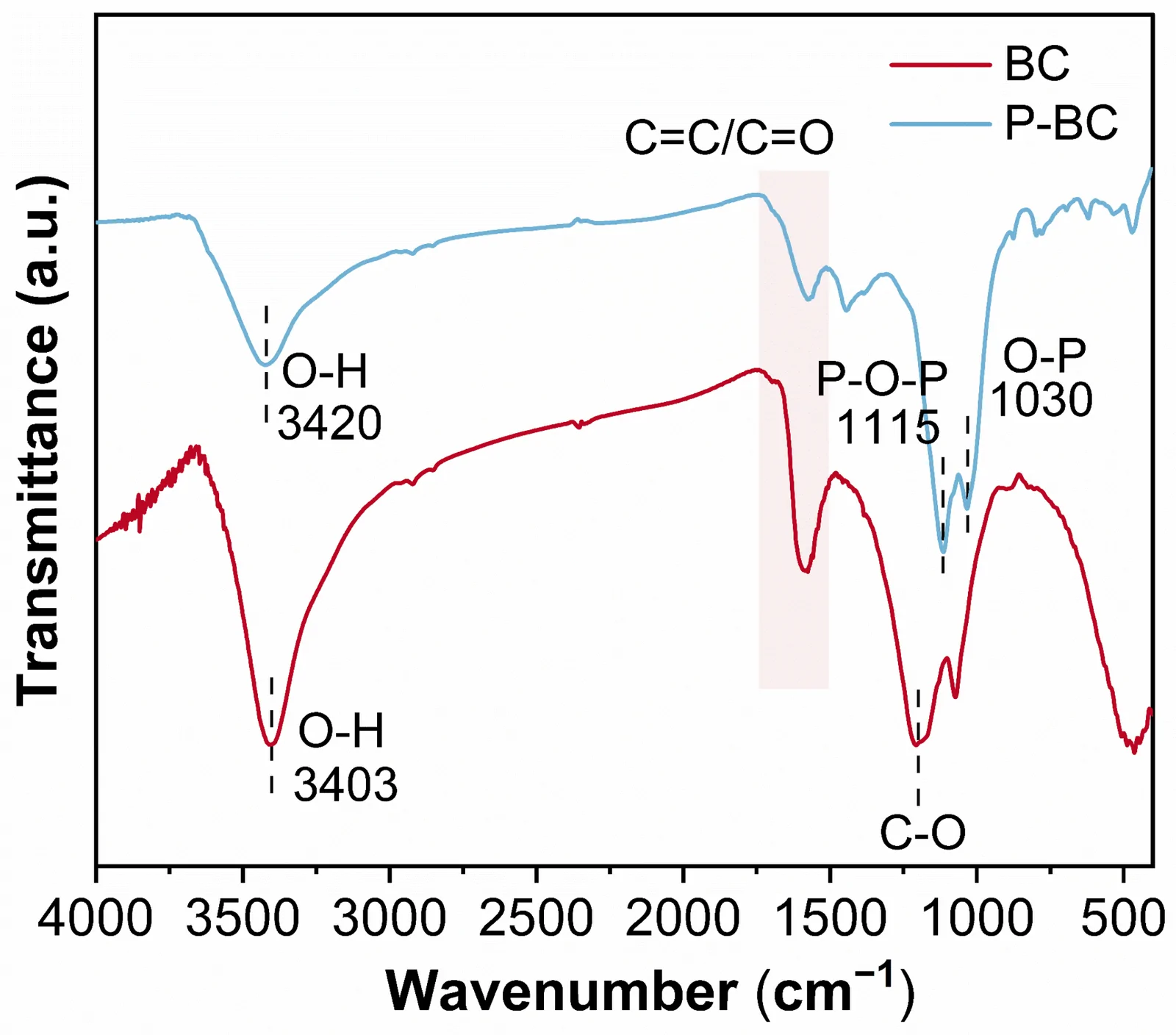
Fourier transform infrared spectroscopy (FTIR) spectra of pristine biochar (BC) and phosphoric acid–modified biochar (P-BC) recorded over the wavenumber range of 4000–400 cm^−1^.

FTIR spectra showed that phosphoric acid modification altered the surface functional-group environment of the wood-derived biochar (Figure 3). The broad O–H stretching band of BC, centered at approximately 3403 cm^−1^, shifted to around 3420 cm^−1^ after modification and decreased in intensity. This change suggests that the chemical environment of surface hydroxyl groups and associated hydrogen-bonding interactions was altered during treatment. Changes were also observed in bands related to the carbon framework. The feature near 1585 cm^−1^, assigned mainly to aromatic C = C vibrations, shifted to approximately 1575 cm^−1^ in P-BC, while the band associated with conjugated C = O changed slightly from about 1697 to 1695 cm^−1^ and became less intense. The persistence of these bands suggests that the aromatic carbon framework was largely retained, although its local chemical environment was modified.

More importantly, P-BC exhibited distinct spectral features near 1115 and 1030 cm^−1^ that were much less evident in pristine BC. These bands are consistent with vibrations associated with phosphate-related P–O–P and P–O structures, providing spectroscopic evidence for the incorporation of phosphorus-containing functionalities after modification. Because vibrations of C–O-containing groups may overlap within this spectral region, however, the assignment of specific phosphorus bonding configurations cannot be established from FTIR alone. These features should therefore be considered together with the subsequent P 2p XPS analysis of P-BC.

The EDS elemental maps were used qualitatively to examine the presence and spatial distribution of phosphorus within the analyzed regions (Figure 1b–d,f–h), rather than as a quantitative measure of phosphorus loading. In P-BC, P signals were distributed across the mapped area without obvious localized aggregation at the spatial resolution of the analysis. Together with the phosphate-related FTIR features, these observations support the presence of phosphorus-containing species after modification.

Taken together, the FTIR and EDS results support the introduction of phosphorus-containing species after modification, while the chemical states of phosphorus in the near-surface region are examined further by XPS below.

#### 2.1.4. Surface Chemical Composition and Bonding States

XPS analysis further confirmed that phosphoric acid treatment substantially altered the surface chemical environment of the wood biochar. The surface atomic fraction of O increased markedly from 14.5% in BC to 31.0% in P-BC, indicating that H_3_PO_4_ modification produced a considerably more oxygen-rich surface. This pronounced increase in surface O suggests a substantial enrichment of oxygen-containing functional groups after modification, consistent with the changes in surface functionality observed by FTIR.

**Figure 4:**
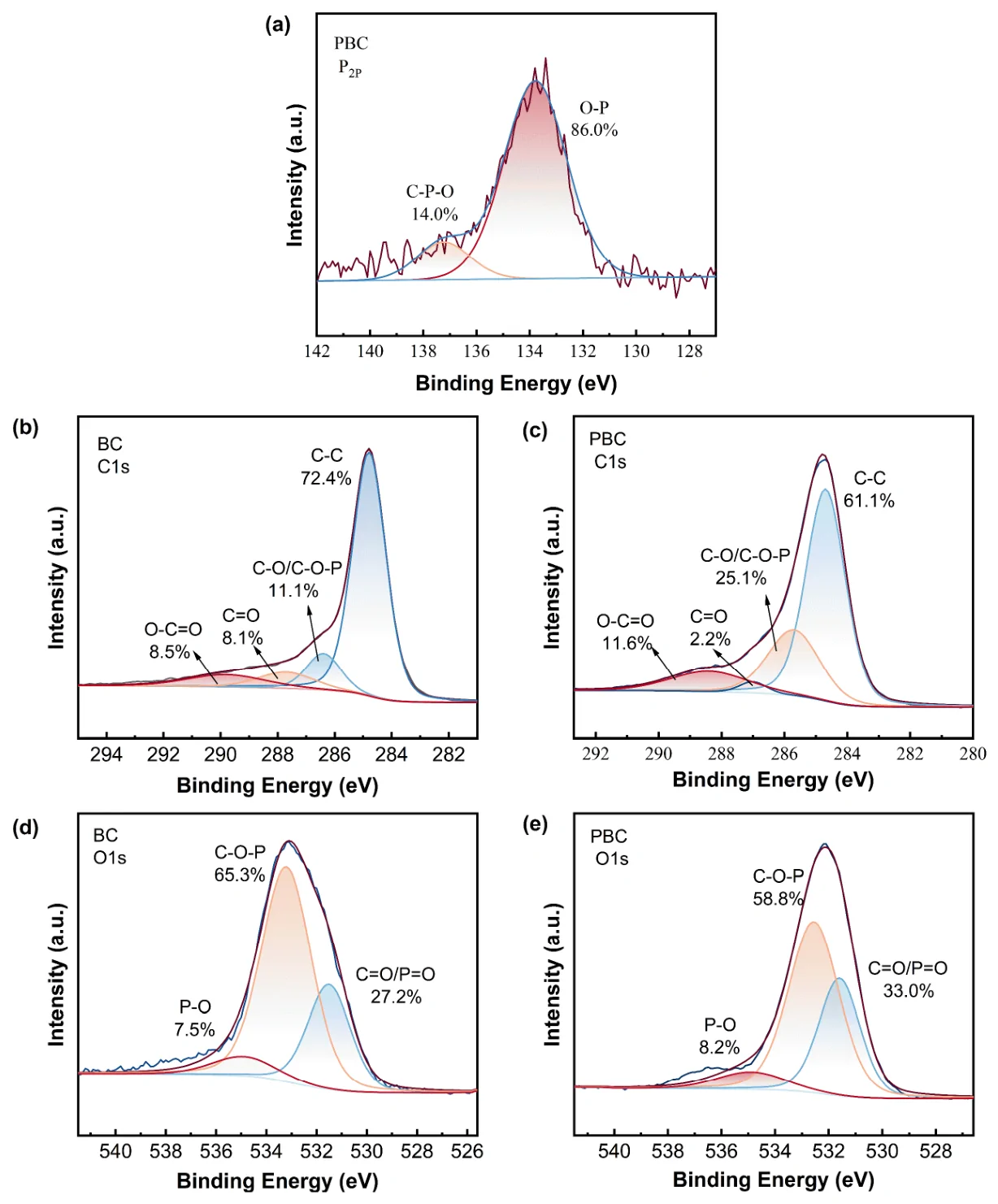
High-resolution XPS spectra of BC and P-BC before Cr(VI) exposure: (**a**) P 2p spectrum of P-BC; (**b**,**c**) C 1s spectra of BC and P-BC; and (**d**,**e**) O 1s spectra of BC and P-BC, respectively. Fitted components indicate the principal C-, O-, and P-containing surface chemical environments. The BC P 2p spectrum is omitted because its weak signal did not permit reliable deconvolution.

The high-resolution C 1s spectra revealed a redistribution of carbon-containing surface species (Figure 4b,c). For BC, the fitted C 1s components assigned to C–C, C–O/C–O–P, C=O, and O–C=O were centered at 284.8, 286.4, 287.7, and 289.8 eV, respectively. The corresponding charge-corrected components for P-BC were centered at 284.8, 285.7, 287.0, and 288.4 eV, respectively. The C–C contribution decreased from 72.4% in BC to 61.1% in P-BC, while the combined C–O/C–O–P component increased from 11.1% to 25.1%. The proportion assigned to O–C=O also increased from 8.5% to 11.6%. These variations are consistent with enrichment of oxygen-containing functionalities and suggest the possible formation of C–O–P linkages during phosphoric acid treatment [17]. Because the C–O and C–O–P contributions were not separated individually during C 1s fitting, however, the increase in this component cannot be taken as a quantitative measure of C–O–P formation itself.

A similar trend was evident in the O 1s spectra (Figure 4d,e). For BC, the fitted O 1s components were centered at 531.5, 533.2, and 534.9 eV, whereas the corresponding components for P-BC occurred at 531.6, 532.6, and 534.9 eV, respectively. The fitted contribution assigned to C=O/P=O increased from 27.2% in BC to 33.0% in P-BC. Together with the substantial increase in the overall surface O content, this shift further demonstrates that phosphoric acid treatment enriched oxygen-containing functional groups and altered the oxygenated surface chemical environment of the biochar. The assignment should nevertheless remain conservative, since C=O and P=O signals overlap within this fitted component. Their individual contributions cannot be resolved from the O 1s spectra alone and are therefore better interpreted alongside the P 2p spectrum of P-BC.

The P 2p signal of pristine BC was weak and did not permit reliable peak deconvolution; therefore, only the P 2p spectrum of P-BC is presented in Figure 4a. The P-BC P 2p spectrum displayed two reproducible fitted components centered at 133.8 and 137.3 eV, indicating the presence of chemically distinct phosphorus-containing environments in the near-surface region. The lower-binding-energy component was consistent with O–P-containing species, whereas the higher-binding-energy component was attributed to a more strongly oxidized or electronically distinct P-containing environment, including possible C–O–P-related structures. These assignments are interpreted cautiously because XPS alone does not uniquely resolve the detailed local bonding configuration of phosphorus. This finding suggests that phosphoric acid treatment altered the bonding state of surface phosphorus and favored its occurrence within oxygen-containing structures. Considered together with the phosphate-related FTIR features and the spatially distributed P signal observed by EDS mapping, the XPS results support a coherent picture in which phosphoric acid modification reorganized the surface functional-group environment and promoted the formation of oxygen-containing phosphorus species.

These surface-chemical changes are also consistent with the altered charge characteristics of P-BC. The pronounced enrichment of oxygenated functionalities, together with the presence of phosphorus-containing surface structures, provides a reasonable chemical basis for the lower pH_pzc_ and the more negative zeta potentials measured after modification. The XPS measurements in the present study were performed before Cr(VI) exposure and therefore establish the initial surface-chemical differences between BC and P-BC. In contrast, our previous H_2_O_2_-functionalization study included post-contact Cr 2p XPS, which provided surface-sensitive Cr-speciation evidence [16]. Accordingly, the XPS results presented here are interpreted specifically in terms of modification-induced interfacial chemistry and are not used to assign the oxidation state or binding environment of retained Cr or to infer a specific post-contact reaction pathway.

#### 2.1.5. Surface-Charge Characteristics and Acid–Base Behavior

**Figure 5:**
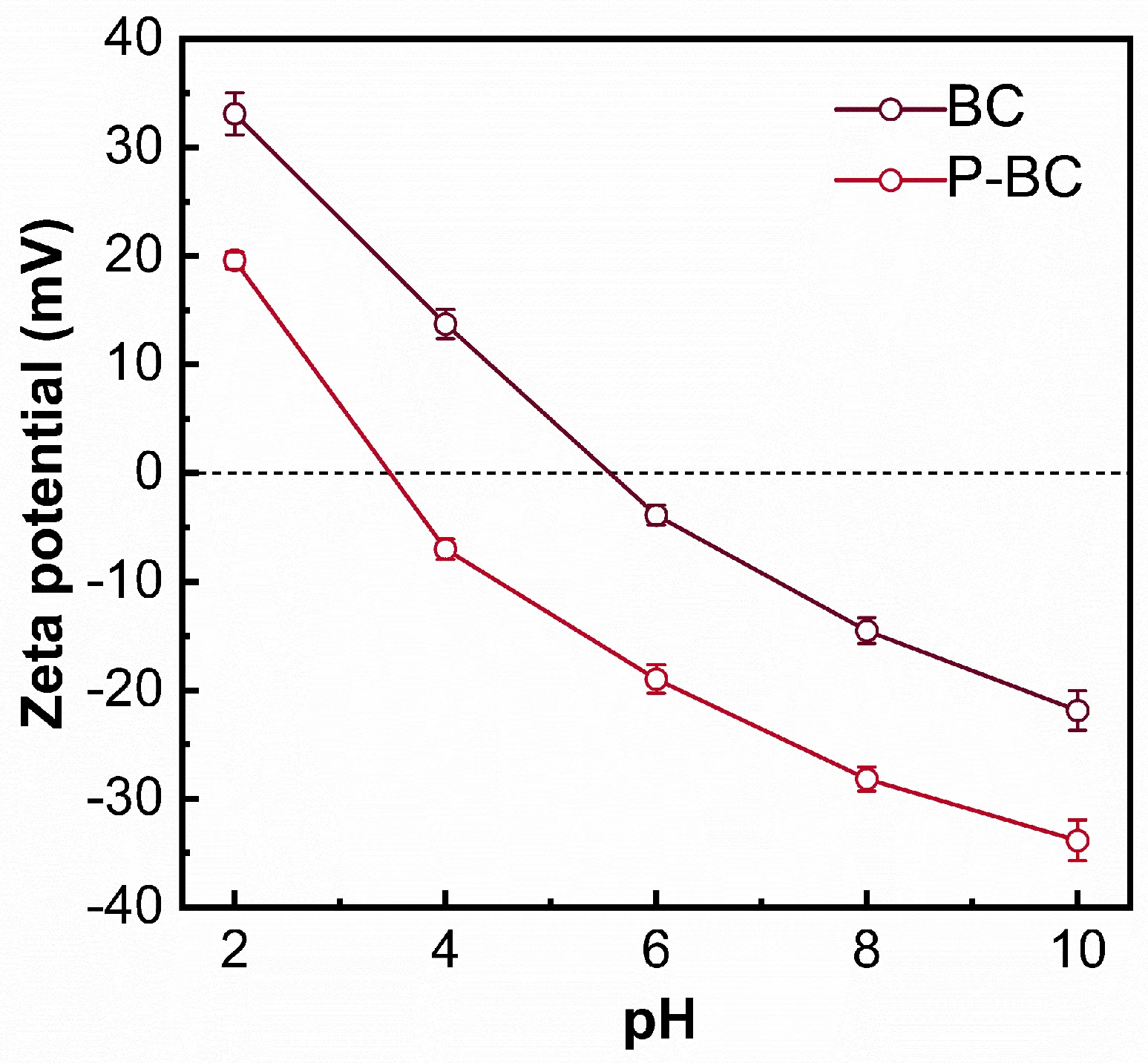
Zeta potential of BC and P-BC as a function of solution pH. Measurements used 0.5 mg mL^−1^ suspensions at pH 2, 4, 6, 8, and 10 after 5 min ultrasonication. Data are mean ± SD (*n* = 3); the dashed line denotes zero zeta potential.

The pH_pzc_ of pristine BC was approximately 5.75, whereas phosphoric acid modification lowered this value to about 3.49, indicating a clear shift toward more acidic surface characteristics. This difference was also reflected in the zeta-potential profiles. BC exhibited positive zeta potentials at pH 2 and 4 and became negatively charged between pH 6 and 10. In comparison, P-BC showed a positive zeta potential only at pH 2 and remained negatively charged from pH 4 to 10. For both materials, zeta potential decreased progressively as solution pH increased, with P-BC displaying consistently more negative values across the investigated range (Figure 5).

The decrease in pH_pzc_ is consistent with the XPS-observed enrichment of oxygen-containing surface functionalities after modification. In particular, the marked increase in surface O, together with the greater contributions of C–O/C–O–P and oxygenated O 1s components, indicates that phosphoric acid treatment produced a more oxygen-rich and chemically heterogeneous surface. The spatially distributed P signal observed by EDS mapping, together with the phosphate-related FTIR features, further supports the presence of phosphorus-containing surface species after modification. These modification-induced changes provide a consistent explanation for the shift in surface acid–base character. Similar shifts toward lower pH_pzc_ values have been reported for phosphoric acid–modified biochars and are associated with changes in acidic surface functionalities [17]. Overall, phosphoric acid modification produced a substantial change in the pH-dependent electrostatic properties of the wood-derived biochar. The implications of these surface-charge characteristics for Cr(VI) retention are discussed in Section 2.2.

### 2.2. pH-Dependent Aqueous Cr(VI)-Derived Retention

These experiments were designed as a focused material-level assessment of pH sensitivity around the measured pH_pzc_ of P-BC (~3.49), rather than to reproduce the pH conditions of the experimental sandy-loam soil. The latter had an initial pH of 6.5 and was used in the subsequent dynamic soil experiments, which provide the more application-oriented assessment of Cr(VI) mobility control. Accordingly, the acidic-range results are interpreted as evidence of pH-dependent interfacial behavior and are not directly extrapolated to circumneutral soil conditions. Across this independently adjusted acidic pH series, both BC and P-BC maintained high Cr(VI) removal efficiencies. P-BC showed slightly higher measured apparent Cr(VI)-derived retention values than BC at each of the three tested pH conditions. Across the tested pH range (pH 2.0–4.0), both BC and P-BC maintained measurable apparent Cr(VI)-derived retention, allowing comparison of pH-dependent retention behavior under controlled aqueous conditions. At pH 2.0, 3.0, and 4.0, the mean apparent equilibrium Cr(VI)-derived retention values for BC were 17.00 ± 0.02, 16.91 ± 0.02, and 16.66 ± 0.02 mg g^−1^, respectively, whereas the corresponding values for P-BC were 17.42 ± 0.01, 17.30 ± 0.03, and 17.18 ± 0.02 mg g^−1^, respectively (mean ± SD, *n* = 3). Thus, P-BC showed slightly higher mean apparent Cr(VI)-derived retention than BC at each tested pH.

Two-way ANOVA indicated significant effects of biochar type (*p* = 1.57 × 10^−14^), initial solution pH (*p* = 1.04 × 10^−10^), and their interaction (*p* = 3.23 × 10^−4^) on apparent Cr(VI)-derived retention. Given the significant interaction, pairwise comparisons were used to evaluate the BC–P-BC difference at each pH. P-BC exhibited significantly higher apparent Cr(VI)-derived retention than BC at pH 2.0, 3.0, and 4.0 (Šidák-adjusted *p* = 1.11 × 10^−4^, 1.50 × 10^−4^, and 4.51 × 10^−5^, respectively). The corresponding mean differences were 0.42, 0.39, and 0.52 mg g^−1^, equivalent to 2.44, 2.28, and 3.15%, respectively. Although these differences were modest in magnitude, they were statistically supported relative to the within-condition variability among independent replicates. Across the tested pH range, mean q_e_ decreased by 2.02% for BC and 1.34% for P-BC.

The overall decrease in mean apparent Cr(VI) retention with increasing pH, together with the modest but statistically supported BC–P-BC differences, can be considered in relation to the surface-charge characteristics described above. Within the investigated acidic range, Cr(VI) occurs predominantly as anionic oxychromate species; therefore, increasingly negative net surface charge would not be expected to enhance retention through simple electrostatic attraction [2,18]. Consistent with this interpretation, at pH 4.0, P-BC still exhibited slightly higher apparent Cr(VI)-derived retention than BC despite its negative zeta potential, indicating that net electrokinetic surface charge alone cannot account for the observed BC–P-BC difference. It should also be noted that zeta potential reflects the net electrokinetic potential at the slipping plane and does not resolve chemically heterogeneous local surface domains or specific interfacial interactions. The persistence of Cr(VI)-derived retention under net-negative surface-charge conditions therefore suggests that non-electrostatic interfacial interactions may also contribute. Specific coordination processes such as ligand exchange or inner-sphere surface complexation are mechanistically plausible, but the present measurements do not directly demonstrate these coordination modes.

Phosphoric acid modification simultaneously altered several material properties, including surface chemistry, N_2_-accessible specific surface area, pore volume, and pore structure. The increased surface O content, enrichment of C–O/C–O–P-related components, incorporation of P-containing species, and enlarged N_2_-accessible surface area collectively demonstrate substantial modification of the interfacial environment. However, none of these pre-exposure measurements identifies the surface sites or reactions directly responsible for Cr(VI)-derived retention. Accordingly, specific surface coordination, surface complexation, and adsorption-coupled reduction remain plausible, non-exclusive pathways, whereas the present data do not permit assignment of any one of these processes as the operative mechanism. The observed differences therefore reflect the pH-dependent response of BC and P-BC within the tested range and should not be interpreted as direct evidence of performance across the full environmental pH spectrum. Because only residual aqueous Cr(VI) was quantified, distinguishing these pathways directly would require post-exposure surface characterization together with independent determination of Cr(VI), Cr(III), and total Cr. In particular, independently replicated retention experiments across approximately pH 5–8 under soil-relevant solution chemistry are needed to determine whether the relative BC–P-BC response observed in the present acidic screening range is maintained under circumneutral conditions. Because the surface-charge characteristics of both materials change substantially with pH, performance at pH 6–8 may differ from that observed at pH 2–4 and therefore cannot be quantitatively inferred from the present dataset.

### 2.3. Soil Water-Retention Performance of the Engineered Amendment

**Figure 6:**
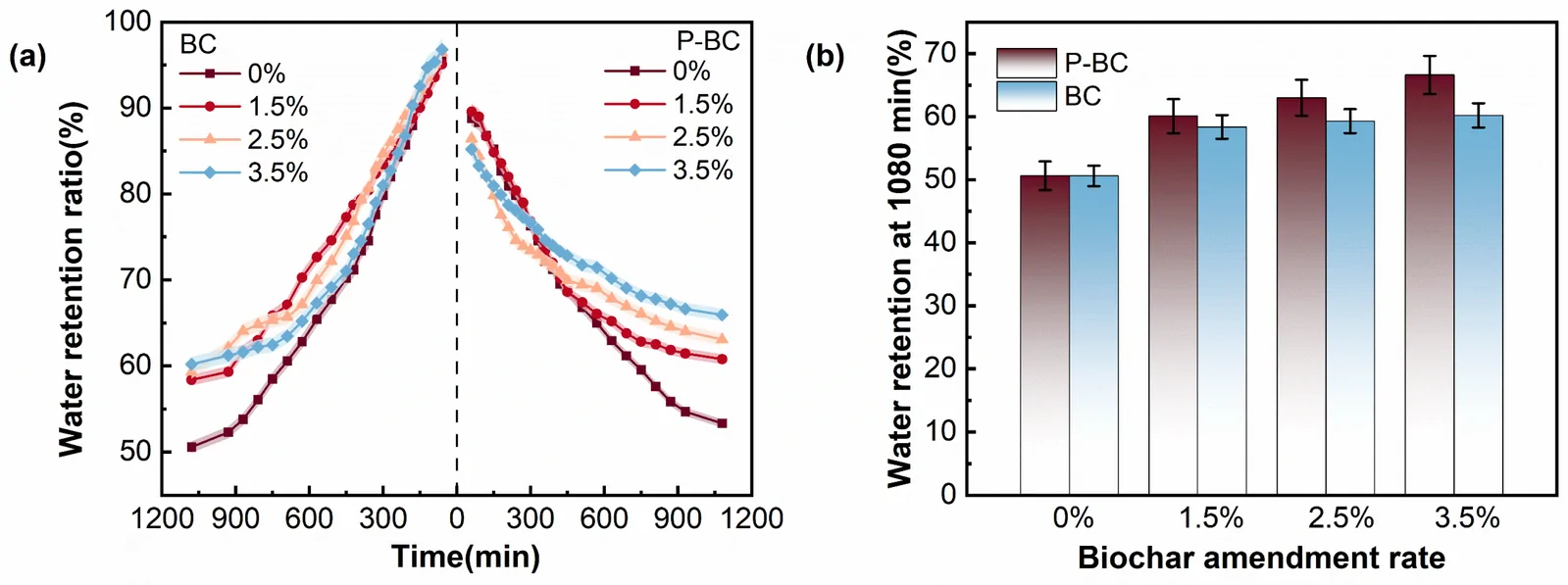
Effects of biochar type and amendment rate on soil water retention during drying. (**a**) Time-dependent residual water-retention ratios for unamended soil (0%) and soils amended with BC or P-BC at 1.5, 2.5, and 3.5% (*w*/*w*). For visual comparison, BC profiles are plotted on a reversed time axis and P-BC profiles on the conventional time axis. (**b**) Residual water-retention ratios after 1080 min. Data are mean ± SD (*n* = 3).

Residual water retention decreased steadily with drying time in all treatments, although the drying trajectories varied with both biochar type and amendment rate (Figure 6a). At 1080 min, the mean residual water-retention ratios for the 1.5, 2.5, and 3.5% P-BC treatments were 59.88 ± 0.52, 63.19 ± 0.26, and 67.15 ± 0.47%, respectively, compared with 59.00 ± 0.56, 59.24 ± 0.38, and 60.11 ± 0.09% for the corresponding BC treatments (mean ± SD, *n* = 3). Two-way ANOVA showed significant effects of biochar type, amendment rate, and their interaction on the 1080-min residual water-retention ratio (all *p* < 0.001). Šidák-adjusted comparisons showed significantly higher values for P-BC than BC at 2.5% (*p* = 0.00073) and 3.5% (*p* = 0.00317), whereas the difference at 1.5% was not statistically supported (*p* = 0.315).

The effect of P-BC on water loss changed over the course of drying rather than remaining constant. At 180 min, the 2.5% and 3.5% P-BC treatments retained 81.46% and 84.44% of their initial water, respectively, both below the 87.96% observed for the unamended control. As drying continued, however, water loss from the P-BC-amended soils slowed. The residual water-retention ratios of the 3.5% and 2.5% treatments exceeded that of the control at approximately 300 and 390 min, respectively (Figure 6a). This crossover suggests that P-BC mainly influenced the rate and timing of water release, giving rise to a stronger retention effect during the later stages of drying rather than suppressing water loss immediately.

Biochar-induced changes in soil water retention can arise from several interacting factors, including amendment rate, soil texture, particle morphology, surface chemistry, and wettability [12]. In the present study, SEM observations showed that P-BC possessed a rougher and more heterogeneous surface, with more pronounced fissures, depressions, and irregular voids than pristine BC. Together with the modification-induced enrichment of oxygen-containing surface functionalities indicated by FTIR and XPS and the phosphorus enrichment revealed by EDS, these morphological differences may alter water–solid interactions and water distribution within the biochar–soil matrix. Because the contribution of individual material properties was not isolated experimentally, the enhanced late-stage water retention of P-BC is most appropriately interpreted as a bulk functional response to the combined effects of morphology and surface chemistry rather than as the consequence of a single structural mechanism. Enhanced water retention alone does not demonstrate Cr(VI) immobilization; its relevance to remediation should therefore be considered together with the subsequent leaching results.

### 2.4. Control of Cr(VI) Migration Under Repeated Infiltration

**Figure 7:**
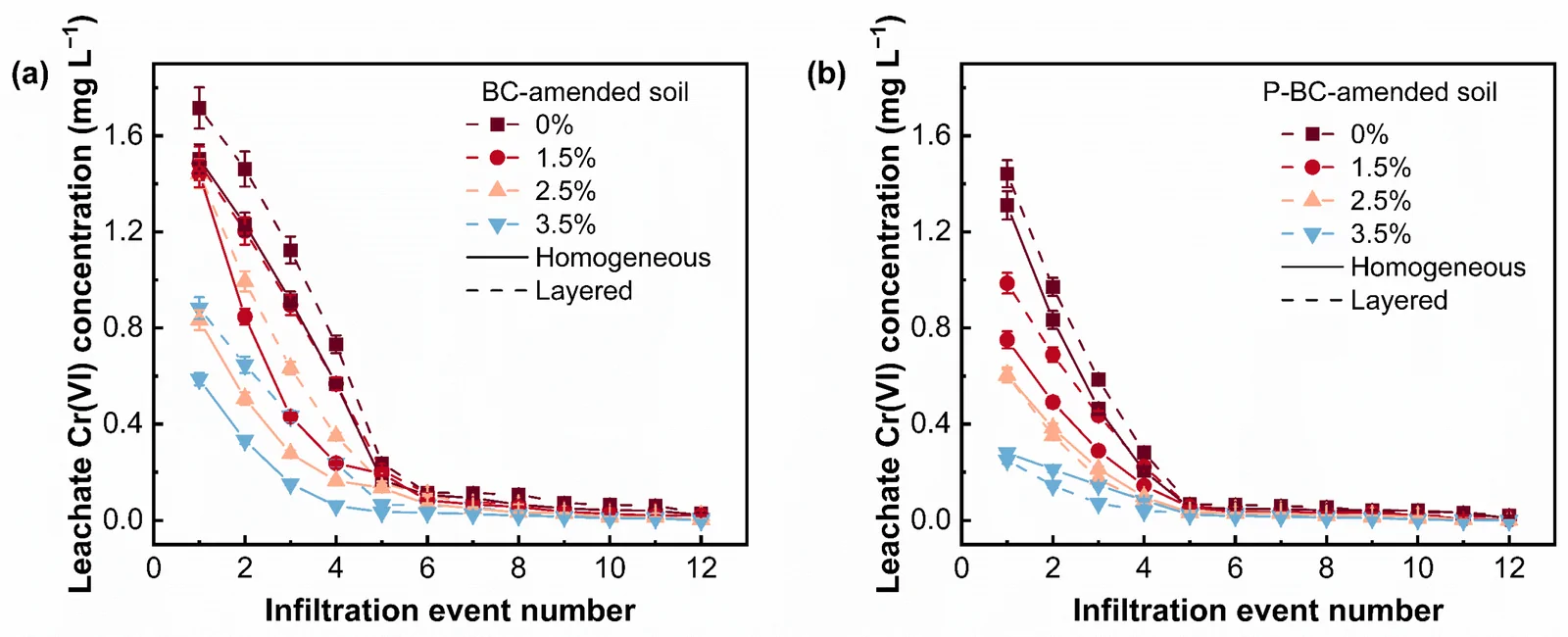
Leachate Cr(VI) concentrations during 12 consecutive infiltration events for soils amended with (**a**) BC and (**b**) P-BC at 0, 1.5, 2.5, and 3.5% (*w*/*w*) using homogeneous or layered placement. Solid and dashed lines denote homogeneous and layered configurations, respectively. Each column received 24 mL of deionized water per infiltration event. Data are mean ± SD of three independent columns (*n* = 3).

Cr(VI) leaching was concentrated primarily in the early stage of the experiment. Across all treatments, 80.8–89.9% of the total Cr(VI) mass leached over 12 days was released within the first four infiltration events. Leachate concentrations then declined sharply, suggesting that the readily mobile Cr(VI) fraction was rapidly depleted before the system entered a slower-release phase (Figure 7a,b). Increasing the biochar application rate consistently lowered cumulative Cr(VI) leaching. For layered BC, cumulative leaching was reduced by 18.3, 33.0, and 57.7% at application rates of 1.5, 2.5, and 3.5%, respectively, relative to the unamended control. The corresponding reductions achieved by homogeneous incorporation were 28.0, 55.7, and 73.4%. Consistent with these cumulative results, the time-resolved profiles showed progressively lower Cr(VI) concentrations in the leachate as the BC application rate increased (Figure 7a). Across the tested application rates, higher BC amendment rates were associated with progressively lower mean cumulative Cr(VI) leaching. This numerical dose-related pattern is consistent with reduced Cr(VI) mobility under repeated infiltration.

The measured P-BC attenuation values varied with spatial configuration and amendment rate. Under layered placement, cumulative Cr(VI) leaching was reduced by 28.7, 61.8, and 82.9% at application rates of 1.5, 2.5, and 3.5%, respectively. When P-BC was incorporated homogeneously, the corresponding reductions were 39.4, 53.6, and 74.1%. At the 1.5% application rate, homogeneous incorporation yielded a lower measured cumulative Cr(VI) leaching value than layered placement, whereas at 2.5 and 3.5% the lower measured values were observed for layered placement (Figure 7b). This crossover pattern suggests that the influence of spatial configuration depended on amendment rate. At the 1.5% application rate, homogeneous incorporation may have increased the probability of contact between the limited P-BC mass and Cr(VI)-bearing pore water throughout the soil matrix. In contrast, at 2.5 and 3.5%, concentrating a larger P-BC mass within a discrete layer may have produced a denser interception zone along the dominant downward flow pathway. Because only three amendment rates were examined, the present results indicate a crossover in configuration response rather than establishing a quantitative threshold. This interpretation is consistent with previous column studies using biochar-based reactive barriers to intercept advective Cr(VI) transport [12].

Mixed-design repeated-measures ANOVA showed significant effects of biochar type, amendment rate, spatial configuration, and infiltration event on the leachate Cr(VI) response, together with significant interaction effects (all *p* < 0.001). In particular, the biochar type × amendment rate × placement interaction was significant (*p* < 0.001), indicating that the effect of spatial configuration depended on both amendment type and application rate. For P-BC, planned comparisons of the 12-event aggregate showed significantly lower leaching under homogeneous incorporation than layered placement at 1.5% (Šidák-adjusted *p* < 0.001), whereas layered placement produced significantly lower leaching at 2.5% (*p* = 0.038) and 3.5% (*p* < 0.001). Thus, the configuration-dependent crossover was statistically supported, although the three tested rates do not establish a quantitative threshold.

Taken together, the measured Cr(VI) attenuation patterns varied with amendment type, application rate, and spatial configuration. The stronger attenuation observed for P-BC coincided with modification-induced changes in surface morphology, pore structure, and phosphorus-containing surface chemistry [7,19]. However, the present experiments do not isolate the contribution of these individual material properties or identify the underlying Cr(VI)-retention pathway. Because only Cr(VI) was quantified in the leachate, the observed decrease is more appropriately described as attenuation of Cr(VI) leaching or apparent Cr(VI) immobilization rather than as direct evidence of adsorption alone [20]. Within these analytical limits, the 3.5% layered P-BC treatment showed the lowest measured cumulative Cr(VI) leaching among the tested configurations.

### 2.5. Persistence of Cr(VI) Attenuation Under Drying–Rehumidification Cycles

Water-extractable Cr(VI) declined progressively over the 12-cycle experiment in all treatments, with treatment trajectories depending on biochar type and amendment rate. Three-way between-unit factorial ANOVA showed significant effects of biochar type and amendment rate, a significant biochar type × amendment rate interaction, and significant effects involving cycle (all cycle-related effects *p* < 0.001).

**Figure 8:**
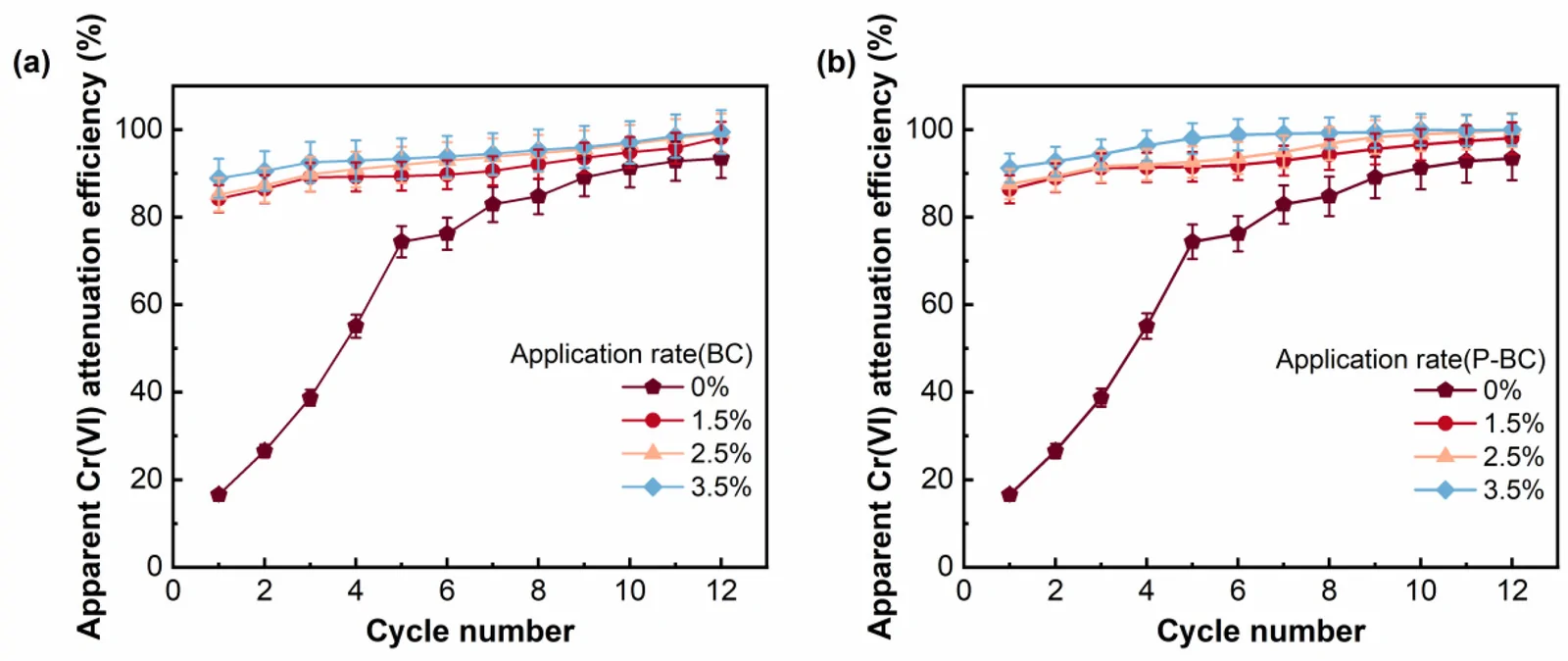
Apparent Cr(VI) attenuation efficiency during repeated drying–rehumidification cycles in soils amended with (**a**) BC and (**b**) P-BC at 0, 1.5, 2.5, and 3.5% (*w*/*w*). Each cycle comprised 12 h drying at 60 °C followed by 12 h rehumidification at 25 °C and 95% relative humidity. Attenuation efficiency (*R*_e_) was calculated relative to the common pre-amendment water-extractable Cr(VI) concentration (*C*_0_ = 15.23 mg L^−1^). Data are mean ± SD (*n* = 3).

At cycle 12, the mean water-extractable Cr(VI) concentrations for the 1.5, 2.5, and 3.5% P-BC treatments were 0.236 ± 0.060, 0.0065 ± 0.0079, and 0.0131 ± 0.0212 mg L^−1^, respectively, compared with 0.220 ± 0.060, 0.0848 ± 0.0304, and 0.0789 ± 0.0225 mg L^−1^ for the corresponding BC treatments. Although lower mean endpoint concentrations were observed for P-BC at 2.5 and 3.5%, the BC–P-BC differences at individual amendment rates were not statistically significant after Šidák correction (adjusted *p* = 0.986, 0.114, and 0.062 at 1.5, 2.5, and 3.5%, respectively). Accordingly, the inferential evidence is stronger for differences in the overall cycling trajectories than for treatment separation at the final cycle alone (Figure 8a,b).

The observed differences between P-BC and BC occurred together with modification-induced changes in surface morphology, pore structure, and surface chemistry [7,19]; however, the present data do not permit these material changes to be linked quantitatively to any individual Cr(VI)-attenuation mechanism. Importantly, water-extractable Cr(VI) also declined markedly in the unamended soil, with apparent attenuation increasing from 16.55% after the first cycle to 93.42% after the twelfth cycle (Figure 8a,b). This background decline indicates that biochar amendment was not the sole process responsible for Cr(VI) loss. Soil minerals, organic matter, Fe-bearing phases, and microbial activity can all contribute to Cr(VI) adsorption, reduction, and stabilization [1,21,22,23], while repeated drying–wetting may further alter Cr mobility and the long-term behavior of remediated soils [13,14,15]. P-BC amendment was associated with lower measured water-extractable Cr(VI) concentrations, particularly at application rates of 2.5 and 3.5%, rather than being regarded as the sole factor governing the observed decline. Because total Cr and Cr(III) were not quantified independently, the decrease is more appropriately described as Cr(VI) attenuation or apparent immobilization and should not be attributed exclusively to either adsorption or reduction [20]. The accelerated cycling conditions should therefore be interpreted as a stress test rather than as a direct simulation of field temperature–moisture regimes. In particular, repeated drying at 60 °C may influence soil organic-matter availability, mineral-surface properties, redox conditions, and microbial activity, thereby contributing to changes in water-extractable Cr(VI) independently of biochar amendment [13,14,15,21,22,23]. Because post-cycle Cr speciation and corresponding soil-phase changes were not independently characterized, the present results do not identify the process responsible for this background attenuation. Future work should evaluate P-BC under field-relevant temperature–moisture regimes together with Cr-speciation-resolved measurements.

### 2.6. Supporting Aqueous-Phase Cr(VI) Retention Kinetics

The aqueous kinetic experiments were intended to complement, rather than serve as the primary basis for, the assessment of soil-scale performance. Apparent Cr(VI)-derived retention increased rapidly for both materials at the beginning of the experiment and then approached a plateau progressively. Within the first 5 min, BC and P-BC reached mean apparent retention values of 6.20 ± 0.07 and 7.19 ± 0.05 mg g^−1^, equivalent to 73.3 and 71.2% of their respective 360-min values. By 360 min, the mean apparent retention values reached 8.46 ± 0.03 mg g^−1^ for BC and 10.10 ± 0.10 mg g^−1^ for P-BC (mean ± SD, *n* = 3), corresponding to a 19.4% higher mean value for P-BC relative to BC. Two-way between-unit factorial ANOVA showed significant effects of biochar type and contact time, together with a significant biochar type × time interaction (all *p* < 0.001). At the prespecified 360-min endpoint, P-BC exhibited significantly higher apparent Cr(VI)-derived retention than BC, with a mean difference of 1.64 mg g^−1^ (95% CI: 1.42–1.87 mg g^−1^; *p* = 0.00052). A comparable two-stage pattern, with rapid initial apparent Cr(VI) depletion followed by a slower approach toward an apparent plateau, has also been reported for phosphoric acid–activated biochars [24]. It should be noted that the baseline apparent Cr(VI)-derived retention of pristine BC in the present study is not directly comparable with that reported in our previous H_2_O_2_-functionalization study [16]. Although the two studies used related aqueous experimental frameworks, the pristine BC materials were independently prepared under different pyrolysis histories and were evaluated under different solution conditions. Variations in precursor batch, pyrolysis temperature/residence time, solution pH control, and agitation intensity may influence Cr(VI) speciation, surface interactions, and mass-transfer behavior. Therefore, cross-study differences in kinetic parameters, model-derived capacities, and diffusion-related descriptors should be interpreted as differences between experimental systems rather than as discrepancies in BC performance.

**Figure 9:**
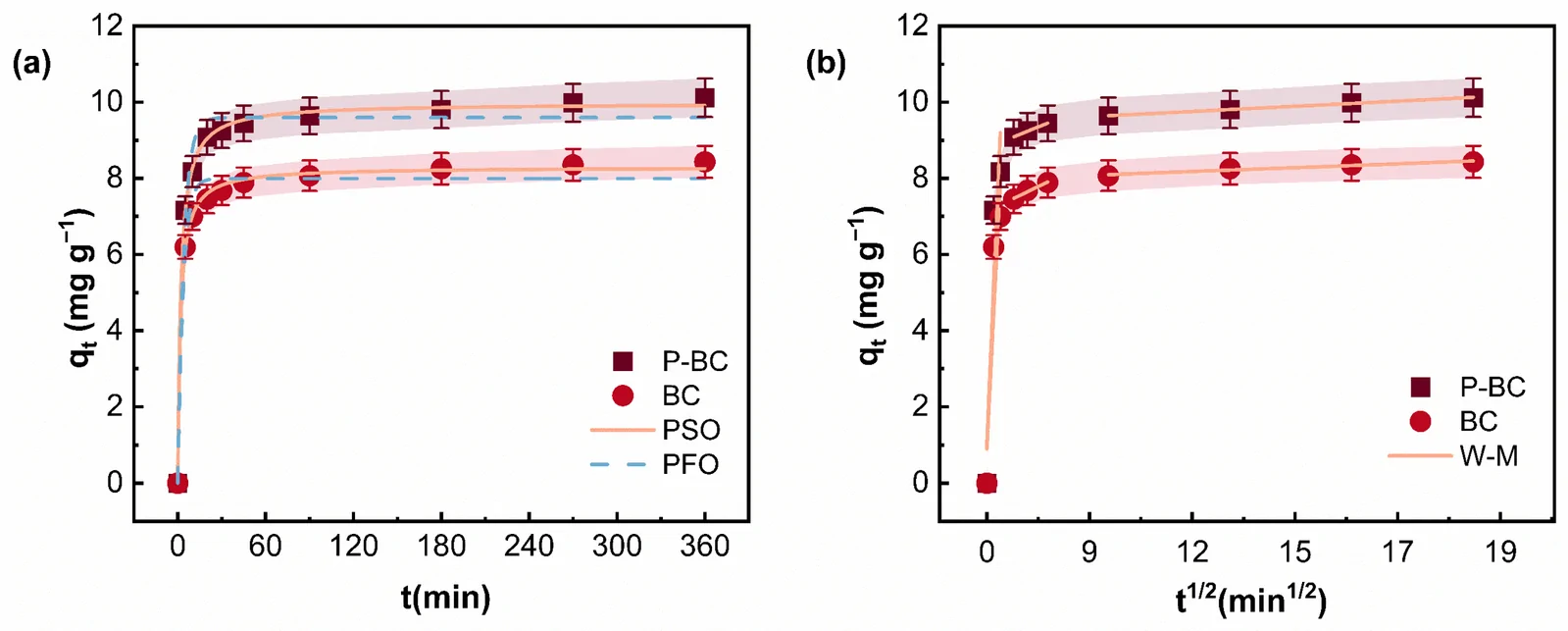
Time-dependent apparent Cr(VI)-derived retention by BC and P-BC. (**a**) Experimental data with pseudo-first-order (PFO) and pseudo-second-order (PSO) fits; (**b**) segmented Weber–Morris q_t_–t^1/2^ plots. Data are mean ± SD (*n* = 3); lines show empirical fits, and shaded bands, where present, indicate 95% confidence intervals of the fitted mean response.

Among the kinetic models examined, the pseudo-second-order (PSO) model provided the closest empirical description of the experimental data, with *R*^2^ values of 0.99805 for BC and 0.99883 for P-BC (Figure 9a). Similar use of empirical kinetic models for describing Cr(VI) removal behavior has been reported in related adsorbent systems [25]. The model-predicted apparent equilibrium retention values differed from the measured 360-min values by only 2.27% for BC and 0.94% for P-BC. Notably, phosphoric acid modification decreased the fitted PSO rate constant, k_2_, from 0.06098 to 0.05152 g mg^−1^ min^−1^, whereas the model-derived initial apparent retention rate, h = k_2_q_e_^2^, increased from approximately 4.14 to 5.18 mg g^−1^ min^−1^. This apparent contrast arises from the mathematical dependence of h on both k_2_ and q_e_: the increase in the fitted q_e_ more than offsets the decrease in k_2_, because q_e_ enters the expression as a squared term. Accordingly, k_2_ and h should not be interpreted independently or assigned direct mechanistic significance; rather, they are retained here as empirical descriptors of the apparent Cr(VI)-depletion kinetics [25,26].

The Weber–Morris representation showed a segmented dependence of q_t_ on t^1/2^ (Figure 9b). The fitted stage-I slope was greater for P-BC than for BC (1.17288 versus 0.38772 mg g^−1^ min^−1/2^), followed by progressively smaller slopes in the later segments. This pattern indicates that the apparent rate of Cr(VI) depletion changed over time, but it does not by itself identify the physical origin of those rate changes. The nonzero intercepts further show that the simple Weber–Morris relation does not pass through the origin. The BET results provide independent structural context for the kinetic observations: relative to BC, P-BC exhibited a larger N_2_-accessible surface area, greater total pore volume, and a larger average pore diameter (Figure 2). These textural changes are consistent with increased pore accessibility but do not establish intraparticle diffusion as the rate-controlling step. Accordingly, the Weber–Morris fit is retained as a descriptive q_t_–t^1/2^ diagnostic rather than direct evidence of a particular mass-transfer mechanism.

### 2.7. Temperature-Dependent Aqueous Cr(VI) Retention

**Figure 10:**
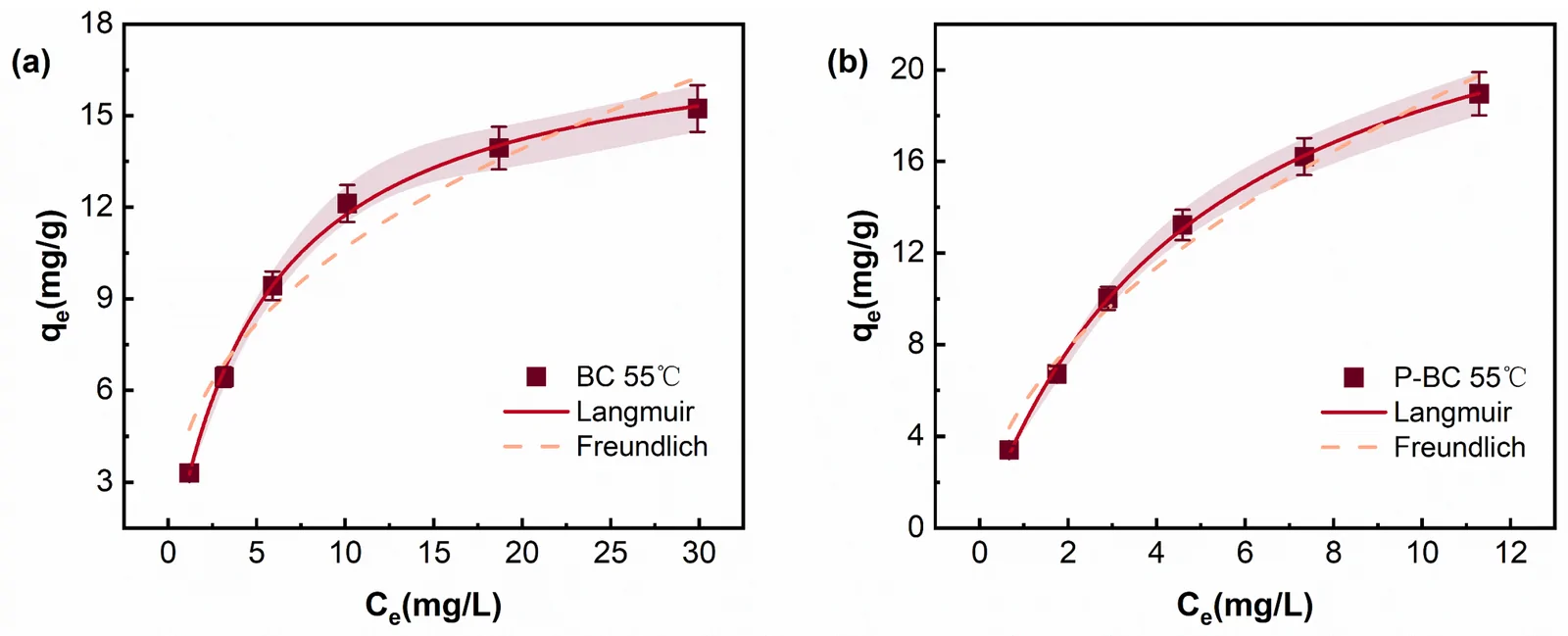
Apparent equilibrium Cr(VI)-derived retention and empirical isotherm fits for (**a**) BC and (**b**) P-BC at 55 °C. Data are mean ± SD (*n* = 3). Solid and dashed lines show Langmuir and Freundlich fits, respectively; shaded bands indicate 95% confidence intervals of the fitted mean response.

Apparent equilibrium Cr(VI)-derived retention increased with both initial Cr(VI) concentration and temperature for BC and P-BC. At an initial Cr(VI) concentration of 106.06 mg L^−1^, the mean apparent equilibrium retention values for P-BC were 10.01 ± 0.01, 15.21 ± 0.19, and 18.96 ± 0.16 mg g^−1^ at 25, 40, and 55 °C, respectively, whereas the corresponding values for BC were 8.71 ± 0.09, 13.01 ± 0.12, and 15.13 ± 0.10 mg g^−1^ (mean ± SD, *n* = 3). The corresponding mean differences between P-BC and BC were 1.30, 2.20, and 3.83 mg g^−1^, equivalent to 14.9, 16.9, and 25.3% relative to BC, respectively. Representative equilibrium isotherms, together with the corresponding Langmuir and Freundlich fits at 55 °C, are presented in Figure 10a,b [19,27]. Three-way ANOVA showed significant effects of biochar type, temperature, and initial Cr(VI) concentration, together with significant two-way and three-way interactions (all *p* < 0.001). Given these significant interactions, prespecified comparisons were performed at *C*_0_ = 106.06 mg L^−1^. P-BC exhibited significantly higher apparent equilibrium Cr(VI)-derived retention than BC at 25, 40, and 55 °C (Šidák-adjusted *p* = 0.0046, 0.00060, and 6.81 × 10^−5^, respectively). It should be noted that these equilibrium tests were conducted at the native, unadjusted pH of the prepared Cr(VI) solutions and therefore are not directly comparable with the independently conducted pH-adjusted screening series in Section 2.2.

The Langmuir-derived apparent maximum retention capacities of P-BC were 12.86, 19.70, and 28.18 mg g^−1^ at 25, 40, and 55 °C, respectively. For BC, the corresponding values were 12.85, 17.77, and 18.08 mg g^−1^. Neither material, however, reached a clearly defined saturation plateau within the concentration range examined. The fitted q_max_ values should therefore be regarded as model-derived apparent retention capacities rather than experimentally verified saturation capacities. Model performance also varied with temperature, and neither the Langmuir nor the Freundlich equation consistently provided the best description across all conditions. Because the fitted q_max_ values are empirical descriptors derived from the present experimental system, they should not be directly compared with values obtained from independently prepared biochars or different solution conditions. From this perspective, differences in fit were treated as empirical features of the equilibrium data rather than as evidence for a particular adsorption mechanism [28]. Representative fits at 55 °C are shown in Figure 10a,b.

The more pronounced temperature response of P-BC is consistent with modification-induced changes in its interfacial properties, although the present data do not establish which surface process is responsible. Apparent equilibrium retention was calculated solely from aqueous Cr(VI) depletion, without independent determination of total Cr or Cr(III). Accordingly, the isotherms describe apparent equilibrium Cr(VI)-derived retention and cannot distinguish among physical retention, redox transformation, and subsequent Cr stabilization. Such processes have been reported for related biochar systems [29,30,31], but their respective contributions are not resolved by the present measurements. These aqueous experiments nevertheless demonstrate that P-BC maintained measurable apparent Cr(VI)-derived retention across the tested operating conditions, whereas the soil experiments provide the more application-relevant evidence of its performance under dynamic environmental conditions.

Taken together, two analytical boundaries should be considered when interpreting the present results. Because the aqueous and soil endpoints in the present study specifically quantify Cr(VI) rather than total Cr or Cr(III), the observed changes are interpreted as apparent Cr(VI)-derived retention and attenuation responses rather than a complete chromium mass balance. In addition, XPS characterization was conducted prior to Cr(VI) exposure and therefore provides evidence of modification-induced changes in the initial surface chemistry rather than direct information on post-contact Cr speciation. Accordingly, the present results establish modification-induced structure–property relationships and enhanced Cr(VI)-attenuation performance, while the relative contributions of adsorption, surface complexation, reduction, and subsequent Cr stabilization remain unresolved.

## 3. Materials and Methods

### 3.1. Reagents and Materials

Analytical-grade potassium dichromate (K_2_Cr_2_O_7_) and phosphoric acid (H_3_PO_4_) were purchased from Shengshi Standard Material Co., Ltd. (Beijing, China) and Sinopharm Chemical Reagent Co., Ltd. (Shanghai, China), respectively. Other chemicals were of analytical grade and used as received. Soil was collected from a semi-arid area in Songpan County, Sichuan Province, China. The collected soil was air-dried at room temperature, gently crushed to break up aggregates, and passed through a 2-mm stainless-steel sieve. The resulting sandy loam had a pH of 6.5 and contained 0.5% organic matter. To prepare Cr(VI)-contaminated soil, an aqueous K_2_Cr_2_O_7_ solution was thoroughly mixed with the untreated soil. The contaminated soil was subsequently homogenized and conditioned at 25 °C for 3 d before further experimentation. The initial water-extractable Cr(VI) concentration of the conditioned contaminated soil, measured before biochar amendment using the extraction procedure described in Section 3.7, was 15.23 mg L^−1^. The contaminated soil was prepared as a controlled laboratory test matrix rather than to reproduce the contamination level of a specific field site. Accordingly, the measured water-extractable Cr(VI) concentration of 15.23 mg L^−1^ was used as the common pre-amendment baseline for comparison among all soil treatments. Because water-extractable Cr(VI) may vary substantially with contamination history, soil properties, and extraction conditions, this value is interpreted as an experimental reference rather than a universal field-site benchmark. Direct numerical comparison with reported field-soil Cr concentrations is not straightforward because many field studies report total soil Cr or Cr(VI) on a mass-per-dry-soil basis, whereas the 15.23 mg L^−1^ value reported here represents an operationally defined water-extractable concentration obtained using the extraction procedure applied in this study.

### 3.2. Preparation of Pristine and Phosphoric Acid–Engineered Biochars

Wood-derived biomass was selected as a lignocellulosic precursor that provides a carbon-rich matrix suitable for high-temperature biochar production and subsequent surface modification [32]. The use of a single wood-derived precursor also enabled a controlled paired comparison between pristine and phosphoric acid–modified biochars prepared from the same feedstock, thereby minimizing feedstock-related variability when evaluating the effects of phosphoric acid treatment. This study was not designed to establish the superiority of wood-derived biochar over biochars produced from other biomass sources. Wood residues were first rinsed thoroughly to remove adhering impurities, then dried and pulverized before pyrolysis. The prepared biomass was pyrolyzed in a tubular furnace under continuous N_2_ flow. The furnace temperature was raised to 750 °C at 5 °C min^−1^ and maintained at this temperature for 2 h. After pyrolysis, the material was allowed to cool to room temperature under the same N_2_ atmosphere [32]. The resulting product was designated pristine biochar (BC).

The pristine BC used in the present study was newly and independently prepared specifically for this experimental series and was not the same batch as that used in our previous H_2_O_2_-functionalization study [16]. The previous study employed a different pyrolysis history (700 °C for 4 h), whereas the present BC was prepared at 750 °C for 2 h. The 750 °C/2 h condition was adopted as the defined preparation protocol for the present H_3_PO_4_-modification series, with reference to the wood-biochar preparation literature cited above [32], rather than to reproduce the pristine BC used in the earlier study. Because pyrolysis temperature and residence time may influence carbonization degree, pore development, surface chemistry, and functional-group abundance, the pristine BC materials from the two studies are not treated as directly equivalent. All modification effects reported in the present work are therefore evaluated by within-study comparison of P-BC with its corresponding pristine BC prepared under the same 750 °C/2 h protocol.

For preparation of P-BC, a 0.4 M H_3_PO_4_ working solution was prepared from a 2.0 M stock solution. Pristine BC was first contacted with the H_3_PO_4_ solution at an initial biochar-to-solution ratio of 1:10 (*w*/*v*). The suspension was subsequently adjusted to an initial pH of 9.0 using 0.1 M NaOH, with 0.1 M HCl used when necessary for fine pH correction, and was then stirred continuously at 25 °C for 72 h. The pH 9.0 value was used as a predefined post-acid-contact conditioning endpoint in the preparation protocol rather than as an environmentally relevant pH or an experimentally optimized synthesis condition. Because the pH adjustment was performed after the initial H_3_PO_4_ contact, the stated 1:10 (*w*/*v*) ratio refers to the initial BC/H_3_PO_4_-solution ratio before titrant addition. At pH 9.0, the dissolved phosphate pool is expected to be predominantly present as deprotonated phosphate species, mainly HPO_4_^2−^, rather than molecular H_3_PO_4_. The treated biochar was subsequently recovered by vacuum filtration and washed repeatedly with ultrapure water until the filtrate approached neutral pH. The washed material was oven-dried at 85 °C and stored in a sealed container until further use. The resulting material was designated phosphoric acid–modified biochar (P-BC) [33].

### 3.3. Physicochemical Characterization

BC and P-BC were characterized prior to Cr(VI) exposure to identify modification-induced changes in surface morphology, surface chemistry, elemental composition, and surface-charge properties. Surface functional groups were examined by Fourier transform infrared spectroscopy (FTIR; Tensor II, Bruker, Ettlingen, Germany) over the wavenumber range of 4000–400 cm^−1^. Spectral changes associated with hydroxyl, carbonyl, aromatic, C–O, and phosphate-related functionalities were compared between BC and P-BC [18,34].

Surface morphology and elemental distributions were examined by scanning electron microscopy coupled with energy-dispersive X-ray spectroscopy (SEM–EDS; Phenom ProX, Thermo Fisher Scientific, Waltham, MA, USA). Images acquired at comparable magnifications were used to compare surface texture, fissures, depressions, and visible pore features. EDS mapping was used qualitatively to examine the spatial distributions of C, O, and P, with particular attention to the presence and within-region distribution of P after modification; the EDS signal was not used as a precise quantitative measure of phosphorus loading.

Textural properties of BC and P-BC were characterized by N_2_ adsorption–desorption measurements at 77.35 K using a BELSORP-max II surface-area and porosity analyzer (MicrotracBEL Corp., Osaka, Japan). The specific surface area was calculated using the Brunauer–Emmett–Teller (BET) method. BET-derived specific surface areas were treated as comparative descriptors of N_2_-accessible surface area and were reported with appropriately limited numerical precision to avoid implying accuracy beyond that supported by the method. Mesopore-size distributions were obtained from the N_2_ adsorption–desorption data using the Barrett–Joyner–Halenda (BJH) procedure implemented in the instrument software. Because BJH analysis is not appropriate for resolving micropore-size distributions, the resulting profiles were interpreted only for mesopore-range features and were not used to characterize pores below 2 nm. Total pore volume and average pore diameter were obtained from the corresponding adsorption–desorption data using the analysis procedures implemented in the instrument software.

Surface elemental composition and chemical states were further characterized by X-ray photoelectron spectroscopy (XPS; ESCALAB, Thermo Fisher Scientific, Waltham, MA, USA). Survey spectra were collected for both materials. High-resolution C 1s, O 1s, and P 2p spectra were acquired for both BC and P-BC. Because the P 2p signal of BC was too weak for reliable peak deconvolution, only the C 1s and O 1s spectra of BC and the C 1s, O 1s, and P 2p spectra of P-BC are presented in Figure 4. Peak deconvolution was performed to evaluate modification-induced changes in C-, O-, and P-containing surface species [18]. Binding energies were charge-corrected by referencing the main C 1s C–C component of each sample to 284.8 eV. High-resolution P 2p spectra were evaluated to identify modification-induced phosphorus-containing surface environments. The fitted P 2p features were interpreted cautiously as chemically distinct P-containing surface environments. The XPS measurements in the present study were conducted prior to Cr(VI) exposure to characterize modification-induced differences in the initial surface chemistry. Accordingly, their interpretation is restricted to pre-exposure interfacial properties and does not extend to post-contact Cr oxidation-state or binding-environment assignment.

The point of zero charge (pH_pzc_) of BC and P-BC was determined using the pH-drift method at a biochar-to-deionized-water mass ratio of 1:20. For zeta-potential measurements, BC and P-BC were dispersed in deionized water at a concentration of 0.5 mg mL^−1^. The suspension pH was adjusted to 2, 4, 6, 8, or 10, followed by ultrasonication for 5 min before measurement. The combined pH_pzc_ and zeta-potential results were used to evaluate modification-induced changes in surface acid–base behavior and pH-dependent electrostatic properties.

### 3.4. Aqueous-Phase Cr(VI) Retention Experiments

Batch Cr(VI) retention tests were performed in Erlenmeyer flasks containing 0.50 g of BC or P-BC and 100 mL of Cr(VI) solution. This solid-to-liquid ratio was selected as a standardized comparative condition to provide sufficient biochar–solution contact while maintaining measurable Cr(VI) concentration changes during kinetic and equilibrium experiments. The same dosage was consistently applied to BC and P-BC to enable direct comparison of modification-induced effects. Unless specified otherwise, the suspensions were shaken at 100 rpm in a temperature-controlled incubator. For the general kinetic and equilibrium experiments, the initial solution pH was not externally adjusted, and the experiments were conducted at the native pH of the prepared Cr(VI) solutions. These general kinetic and equilibrium tests were conducted as an experimental series independent of the deliberately pH-adjusted screening experiments described below; therefore, values obtained from the two series should not be interpreted as measurements made under identical pH conditions. For the equilibrium tests, Cr(VI) solutions with initial concentrations of 17.67, 35.35, 53.03, 70.71, 88.38, and 106.06 mg L^−1^ were used. The suspensions were maintained for 6 h at 25, 40, or 55 °C. At the end of equilibration, a 10 mL aliquot was collected and vacuum-filtered prior to Cr(VI) determination. Kinetic tests were conducted separately at 25 °C using an initial Cr(VI) concentration of 106.06 mg L^−1^. Separate sacrificial flasks were prepared for each sampling time (0, 5, 10, 20, 30, 45, 90, 180, 270, and 360 min), and the entire corresponding flask was sampled at the designated time and processed using the same filtration procedure [18,35]. The kinetic and equilibrium experiments were each conducted using three independent experimental replicates per condition (*n* = 3). Experimental apparent-retention values are reported as mean ± standard deviation (SD), with replicate-based SD shown as error bars in Figure 9 and Figure 10. All treatments were analyzed using the same DPC colorimetric procedure to maintain analytical consistency across within-study comparisons. The independent experimental replicates characterize experimental variability and are not regarded as a substitute for dedicated analytical quality-control measurements. Certified reference materials and a separate trace-level analytical validation procedure were not included in the present experimental design. Accordingly, the Cr(VI) measurements are interpreted primarily for relative comparison among treatments analyzed using the same procedure, and the lowest measured endpoint concentrations are not used to support high-precision trace-level quantification.

The effect of initial solution pH was evaluated in an independent acidic-range screening series at pH 2.0, 3.0, and 4.0. This interval was selected as a focused material-level pH-response window spanning the measured pH_pzc_ of P-BC (approximately 3.49) and the observed change in zeta-potential sign between pH 2 and 4. The purpose of this series was therefore to examine pH-sensitive interfacial behavior near this charge-transition region rather than to survey the broader environmental pH range. Accordingly, circumneutral ranges such as pH 5–8, although more representative of many soils, address a different application-oriented question and were not included in this focused pH-response screening design. For each pH condition, 0.50 g of BC or P-BC was mixed with 100 mL of Cr(VI) solution (*C*_0_ = 106.06 mg L^−1^) and reacted for 6 h at 25 °C. The initial pH was adjusted with 0.1 M HCl or NaOH as required. After treatment, the residual Cr(VI) concentration in the filtrate was determined at 540 nm using the 1,5-diphenylcarbazide (DPC) colorimetric method [36]. Each biochar–pH condition was independently replicated three times (*n* = 3), and the resulting apparent Cr(VI)-derived retention values are reported as mean ± standard deviation (SD). Replicate-level measurements were used for statistical comparisons between BC and P-BC, as described in Section 3.8.

The apparent Cr(VI) depletion-derived retention quantities at equilibrium (q_e_, mg g^−1^) and at time t(q_t_, mg g^−1^) were calculated from aqueous-phase Cr(VI) mass balances:

(1) $$q_{e}=\frac{\left(C_{0}-C_{e}\right)\times V}{m}$$

(2) $$q_{t}=\frac{\left(C_{0}-C_{t}\right)\times V}{m}$$

where *C*_0_, *C*_e_, and *C*_t_ (mg L^−1^) denote the initial, equilibrium, and time-dependent Cr(VI) concentrations, respectively; V (L) is the solution volume; and m (g) is the biochar mass. Because q_e_ and q_t_ are derived solely from the decrease in dissolved Cr(VI), they represent operational measures of apparent Cr(VI)-derived retention and should not be interpreted as independently measured solid-phase adsorption capacities. For clarity, aqueous q_e_ and q_t_ values are described throughout as apparent Cr(VI)-derived retention, whereas decreases in leachate or water-extractable Cr(VI) in soil experiments are described as Cr(VI) attenuation or apparent immobilization; adsorption is not inferred from Cr(VI) depletion alone.

Equilibrium data were described empirically using the Langmuir and Freundlich isotherm models [35]:

(3) $$q_{e}=\frac{q_{\max}K_{L}C_{e}}{1+K_{L}C_{e}}$$

(4) $$q_{e}=K_{F}C_{e}^{1/n}$$

where q_max_ (mg g^−1^) is the Langmuir-derived apparent maximum retention capacity, *K*_L_ (L mg^−1^) is the empirical Langmuir coefficient, *K*_F_ is the empirical Freundlich capacity coefficient, and *n* is the Freundlich heterogeneity exponent. These fitted parameters are used descriptively and are not assigned direct mechanistic meaning.

Time-dependent apparent Cr(VI)-derived retention data were evaluated empirically using the pseudo-first-order (PFO), pseudo-second-order (PSO), and Weber–Morris t^1/2^ formulations [35]:

(5) $$q_{t}=q_{e}\left(1-e^{-k_{1}t}\right)$$

(6) $$q_{t}=\frac{k_{2}q_{e}^{2}t}{1+k_{2}q_{e}t}$$

(7) $$q_{t}=k_{p}t^{1/2}+C$$

where k_1_ and k_2_ are empirical PFO and PSO rate parameters, respectively; k_p_ is the slope of the Weber–Morris q_t_–t^1/2^ segment; and *C* is the corresponding intercept. Model performance was assessed using the agreement between the fitted curves and experimental data. For the independently replicated kinetic and equilibrium datasets, experimental uncertainty is represented by mean ± SD (*n* = 3), with SD shown as error bars in Figure 9 and Figure 10. The shaded bands represent 95% confidence intervals of the nonlinear fitted mean response and are distinct from replicate-based experimental SD. Because all q_e_ and q_t_ values are derived solely from dissolved Cr(VI) depletion, the fitted kinetic and equilibrium models are used as empirical descriptors of apparent Cr(VI)-derived retention rather than as evidence for adsorption, chemisorption, intraparticle-diffusion control, reduction, or any other unique molecular pathway [26,28].

### 3.5. Soil Water-Retention Experiment

Soil water-retention behavior was evaluated using uncontaminated soil [9,10]. For each treatment, 150 g of soil was thoroughly mixed with BC or P-BC at application rates of 0, 1.5, 2.5, or 3.5% (*w*/*w*, on a dry-soil mass basis). All treatments were adjusted to the same initial moisture content and placed at equal distances from a radiant heat source to maintain comparable thermal exposure. Throughout the experiment, the ambient temperature and relative humidity were maintained at 25 °C and 45%, respectively. Each treatment was performed in triplicate, and the results are presented as mean ± standard deviation (SD).

Water loss was monitored gravimetrically during drying. Sample masses were recorded at 0, 10, 20, and 30 min, followed by measurements at 30-min intervals during the intermediate drying stage and at progressively longer intervals thereafter. The final measurement was taken at 1080 min. The residual water-retention ratio, *R*_w_ (%), was calculated as:

(8) $$R_{w}=\frac{M_{t}-M_{d}}{M_{0}-M_{d}}\times 100$$

where *M*_0_ and *M*_t_(g) are the initial total mass and the total mass at time t, respectively, and *M*_d_ (g) is the dry mass of the soil–biochar mixture. Thus, *M*_0_ − *M*_d_ and *M*_t_ − *M*_d_ represent the initial and residual water masses.

### 3.6. Repeated-Infiltration Soil-Column Experiment

Cr(VI) transport under repeated infiltration was investigated using columns packed with 200 g of Cr(VI)-contaminated soil. The experimental treatments included an unamended control and soils amended with BC or P-BC at 1.5, 2.5, or 3.5% (*w*/*w*, based on dry soil mass). To examine whether amendment placement affected Cr(VI) migration, two configurations were compared. In the homogeneous treatment, the required biochar dose was thoroughly mixed with the entire 200 g soil matrix. For the layered treatment, 100 g of contaminated soil was first placed in the lower part of the column, followed by the full biochar dose as a distinct intermediate reactive layer; the remaining 100 g of contaminated soil was then added above it. This configuration was designed to represent a localized biochar-based interception zone capable of limiting Cr transport during downward infiltration [12,37].

Each treatment was performed using three independent soil columns, and the results are presented as mean ± standard deviation (SD). Each column received 24 mL of deionized water at 24 h intervals for 12 consecutive days. After each infiltration event, the leachate was collected and its total volume recorded. A 10 mL aliquot was then vacuum-filtered, and the Cr(VI) concentration in the filtrate was determined using the 1,5-diphenylcarbazide (DPC) colorimetric method at 540 nm [36,38].

The cumulative mass of Cr(VI) leached during the experiment, *M*_cum_ (mg), was calculated as:

(9) $$M_{cum}=\sum_{i=1}^{n}\left(C_{i}V_{i}\right)$$

where *M*_cum_ (mg) is the cumulative Cr(VI) mass released during the experiment, *C*_i_ (mg L^−1^) is the Cr(VI) concentration in the leachate collected after the ith infiltration event, *V*_i_ (L) is the corresponding leachate volume, and *n* is the total number of infiltration events.

The effectiveness of each biochar treatment in suppressing Cr(VI) migration was expressed as the cumulative Cr(VI) leaching attenuation efficiency, η (%):

(10) $$\eta =\frac{M_{CK}-M_{treat}}{M_{CK}}\times 100$$

where *M*_CK_ and *M*_treat_ (mg) denote the cumulative Cr(VI) masses released from the unamended control and the corresponding biochar-amended treatment, respectively.

### 3.7. Environmental Stability Under Drying–Rehumidification Cycles

The persistence of Cr(VI) attenuation under repeated hygrothermal stress was evaluated using Cr(VI)-contaminated soil homogeneously amended with BC or P-BC at 1.5, 2.5, or 3.5% (*w*/*w*, based on dry soil mass), together with an unamended control. For each treatment and target sampling cycle, independent 200-g soil units were prepared. Units designated for a given sampling endpoint were subjected to the corresponding number of consecutive 24-h drying–rehumidification cycles and were then sacrificed for determination of water-extractable Cr(VI). Each 24-h cycle comprised 12 h of oven drying at 60 °C followed by 12 h of rehumidification at 25 °C and 95% relative humidity. This protocol was designed as an accelerated hygrothermal stress test to evaluate whether the observed Cr(VI)-attenuation response persisted under repeated drying–rehumidification perturbation; it was not intended to reproduce a specific field temperature–moisture regime [14].

Each treatment–cycle combination was independently replicated three times, and the results are presented as mean ± standard deviation (SD). The measured initial water-extractable Cr(VI) concentration of the contaminated soil before biochar amendment and cyclic treatment was 15.23 mg L^−1^ and was used as the common experimental reference concentration (*C*_0_) for all treatments. Accordingly, the calculated apparent attenuation efficiency represents the overall attenuation relative to the initial contaminated soil and includes the effects associated with biochar amendment and the imposed drying–rehumidification treatment [20]. The sample was mixed with deionized water at a solid-to-liquid ratio of 1:2 (*w*/*v*) and continuously agitated for 2 h. After extraction, the suspension was filtered through a 0.45 μm membrane, and the Cr(VI) concentration in the filtrate was measured using the 1,5-diphenylcarbazide (DPC) colorimetric method at 540 nm [18,38]. The analytical scope and quality-control limitations of the Cr(VI) measurements were as described in Section 3.4.

Because variations in water-extractable Cr(VI) may reflect several concurrent processes, including adsorption, reduction, mineral association, and other transformations, rather than adsorption alone [20], treatment performance was expressed as the apparent Cr(VI) attenuation efficiency, *R*_e_ (%):

(11) $$R_{e}=\frac{C_{0}-C_{t}}{C_{0}}\times 100$$

where *C*_0_ (mg L^−1^) is the measured initial water-extractable Cr(VI) concentration of the contaminated soil before biochar amendment and cyclic treatment (15.23 mg L^−1^), and *C*_t_ (mg L^−1^) is the corresponding water-extractable Cr(VI) concentration measured after cycle t. Thus, *R*_e_ represents the overall apparent Cr(VI) attenuation relative to the common pre-amendment baseline.

### 3.8. Statistical Analysis

Statistical analyses were performed using replicate-level experimental data. Unless otherwise stated, quantitative results are reported as mean ± standard deviation (SD) from three independent experimental replicates per condition (*n* = 3), and statistical significance was defined as *p* < 0.05. For the pH-dependent Cr(VI)-retention experiment, two-way factorial ANOVA was used to evaluate the effects of biochar type, initial solution pH, and their interaction. For the equilibrium experiment, three-way factorial ANOVA was used to evaluate the effects of biochar type, temperature, initial Cr(VI) concentration, and their interactions, with initial concentration treated as a categorical experimental factor.

Because separate sacrificial flasks were used at each kinetic sampling time, the kinetic dataset was analyzed using two-way factorial ANOVA, with biochar type and contact time treated as between-unit factors. Similarly, because independent sacrificial soil units were used for each sampling cycle in the drying–rehumidification experiment, these data were analyzed using three-way factorial ANOVA, with biochar type, amendment rate, and drying–rehumidification cycle treated as between-unit factors. For the repeated-infiltration experiment, repeated measurements were obtained from the same soil columns across successive infiltration events. Accordingly, these data were analyzed using mixed-design repeated-measures ANOVA, with biochar type, amendment rate, and placement configuration treated as between-column factors and infiltration event treated as the within-column factor. Greenhouse–Geisser correction was applied to within-column effects where appropriate to account for departures from sphericity.

The 1080-min soil water-retention endpoint was analyzed using two-way factorial ANOVA with biochar type and amendment rate as fixed factors. The 0% unamended treatments were not included in factorial analyses involving biochar type, amendment rate, or placement configuration because these treatment factors were not defined for unamended soil. Where significant interactions were detected, prespecified pairwise comparisons were performed using two-sided Welch’s t-tests with Šidák adjustment within each family of comparisons. Effect estimates and 95% confidence intervals are reported for the principal BC–P-BC contrasts.

## 4. Conclusions

Phosphoric acid modification altered the external morphology, pore structure, and interfacial chemistry of wood-derived biochar, as evidenced by SEM–EDS, N_2_ adsorption–desorption, FTIR, XPS, and surface-charge measurements. Phosphorus incorporation was accompanied by a marked shift in surface acid–base behavior, with the pH_pzc_ decreasing from approximately 5.75 for BC to 3.49 for P-BC. In aqueous systems, P-BC showed slightly higher mean apparent Cr(VI)-derived retention than BC across the tested pH range of 2.0–4.0. The 360-min mean apparent retention values were 8.46 ± 0.03 mg g^−1^ for BC and 10.10 ± 0.10 mg g^−1^ for P-BC, while the Langmuir-derived apparent maximum retention capacity at 55 °C was 18.08 and 28.18 mg g^−1^, respectively. These results demonstrate that phosphoric acid modification resulted in higher apparent Cr(VI)-derived retention under the tested aqueous conditions, although the specific interfacial process responsible for this difference could not be resolved from the present measurements.

P-BC-amended soils showed higher measured late-stage water-retention values and lower measured Cr(VI) leaching under repeated infiltration. Among the tested treatments, the 3.5% layered P-BC treatment yielded the lowest measured cumulative Cr(VI) leaching, corresponding to an 82.9% reduction relative to the unamended control. This result also highlights the combined importance of amendment rate and spatial placement in determining remediation performance. Inferential analyses further showed that the effects of phosphoric acid modification depended on operating conditions and treatment context, including temperature, amendment rate, spatial placement, infiltration event, and drying–rehumidification cycle. Statistically supported BC–P-BC differences were observed for the kinetic and equilibrium aqueous endpoints, final soil water retention at the higher amendment rates, and repeated-infiltration responses, whereas treatment differences at the final drying–rehumidification cycle were not significant after multiplicity correction.

Overall, phosphoric acid modification was associated with higher apparent aqueous Cr(VI)-derived retention and lower measured Cr(VI) mobility under several tested soil conditions. P-BC therefore warrants further evaluation as an engineered biochar amendment for aqueous Cr(VI) treatment and soil mobility control. Among the configurations examined, layered placement at the higher amendment rates produced lower measured cumulative Cr(VI) leaching; however, the present accelerated experiments do not establish long-term field stability.

## Data Availability

Data will be made available on request.

## References

[1] Ao M., Chen X., Deng T., Sun S., Tang Y., Morel J.L., Qiu R., Wang S. (2022). Chromium Biogeochemical Behaviour in Soil-Plant Systems and Remediation Strategies: A Critical Review. J. Hazard. Mater..

[2] Sinha R., Kumar R., Sharma P., Kant N., Shang J., Aminabhavi T.M. (2022). Removal of hexavalent chromium via biochar-based adsorbents: State-of-the-art, challenges, and future perspectives. J. Environ. Manag..

[3] Liu J., Sun S., Zhang H., Kong Q., Li Q., Yao X. (2023). Remediation Materials for the Immobilization of Hexavalent Chromium in Contaminated Soil: Preparation, Applications, and Mechanisms. Environ. Res..

[4] Zheng C., Yang Z., Si M., Zhu F., Yang W., Zhao F., Shi Y. (2021). Application of Biochars in the Remediation of Chromium Contamination: Fabrication, Mechanisms, and Interfering Species. J. Hazard. Mater..

[5] Ambika S., Kumar M., Pisharody L., Malhotra M., Kumar G., Sreedharan V., Singh L., Nidheesh P.V., Bhatnagar A. (2022). Modified Biochar as a Green Adsorbent for Removal of Hexavalent Chromium from Various Environmental Matrices: Mechanisms, Methods, and Prospects. Chem. Eng. J..

[6] Zhong M., Zhang Q., Li M., Abodif A.M., Ming T., Fan Z., Gao B. (2023). Biochar as a Multifunctional Agent for Aqueous Chromium Removal: A Critical Review of Governing Mechanisms, Targeted Syntheses, Influencing Factors, and Practical Applications. Chem. Eng. J..

[7] Xiao X., Zhou F., Yang C., Lu L., Zhao Y., Tu B., Li L. (2025). Adsorption and Reduction of Cr (VI) by C-P-O Groups in Biochar: Performance and Mechanisms. Solid State Sci..

[8] Chen M., Chen X., Xu X., Xu Z., Zhang Y., Song B., Tsang D.C.W., Xu N., Cao X. (2022). Biochar Colloids Facilitate Transport and Transformation of Cr (VI) in Soil: Active Site Competition Coupling with Reduction Reaction. J. Hazard. Mater..

[9] Wei B., Peng Y., Lin L., Zhang D., Ma L., Jiang L., Li Y., He T. (2023). Drivers of Biochar-Mediated Improvement of Soil Water Retention Capacity Based on Soil Texture: A Meta-Analysis. Geoderma.

[10] Basso A.S., Miguez F.E., Laird D.A., Horton R., Westgate M. (2013). Assessing potential of biochar for increasing water-holding capacity of sandy soils. GCB Bioenergy.

[11] Tang X.-Y., Yin W.-M., Yang G., Cui J.-F., Cheng J.-H., Yang F., Li X.-Y., Wu C.-Y., Zhu S.-G. (2024). Biochar Reduces Antibiotic Transport by Altering Soil Hydrology and Enhancing Antibiotic Sorption. J. Hazard. Mater..

[12] Zhou Z., Liu P., Wang S., Finfrock Y.Z., Ye Z., Feng Y., Li X. (2022). Iron-Modified Biochar-Based Bilayer Permeable Reactive Barrier for Cr(VI) Removal. J. Hazard. Mater..

[13] Jia J., Yao L., Xiao B., Fan X., Wang X., Liu Y., Wu Y., Hu L. (2024). Mechanisms and influential factors of soil chromium long-term stability by an accelerated aging system after chemical stabilization. J. Hazard. Mater..

[14] Xie L., Zhou Y., Ma Q., Zhang J., Duan G., Lin A., Xiao Y. (2025). Long-term effectiveness evaluation of Cr (VI)-contaminated soil remediation with modified iron-biochar composites. J. Environ. Chem. Eng..

[15] Tan X., Qi F., Liu Q., Qie H., Duan G., Lin A., Liu M., Xiao Y. (2024). Is Cr (III) re-oxidation occurring in Cr-contaminated soils after remediation: Meta-analysis and machine learning prediction. J. Hazard. Mater..

[16] Li S., Tang J., Su Z., Ren L., Wu Y., Guo G., Rao J., Zhou M., Fan Y. (2026). Oxidative Functionalization of Woody Biochar for Hexavalent Chromium Detoxification: Adsorption-Coupled Reduction and Dual-Phase Remediation. Molecules.

[17] Zeng X.-Y., Wang Y., Li R.-X., Cao H.-L., Li Y.-F., Lü J. (2022). Impacts of Temperatures and Phosphoric-Acid Modification to the Physicochemical Properties of Biochar for Excellent Sulfadiazine Adsorption. Biochar.

[18] Xie D., Yang J., Zhou X., Song M., Wang X. (2026). Efficient Removal of Cr (VI) Using Low-Temperature Biochar Derived from Stevia Residues via Wet-Process Phosphoric Acid Pretreatment. Environ. Res..

[19] Zeng H., Zeng H., Zhang H., Shahab A., Zhang K., Lu Y., Nabi I., Naseem F., Ullah H. (2021). Efficient Adsorption of Cr (VI) from Aqueous Environments by Phosphoric Acid Activated Eucalyptus Biochar. J. Clean. Prod..

[20] Guo Q., Yu D., Yang J., Zhao T., Yu D., Li L., Wang D. (2024). A Novel Sequential Extraction Method for the Measurement of Cr (VI) and Cr (III) Species Distribution in Soil: New Insights into the Chromium Speciation. J. Hazard. Mater..

[21] Dhal B., Thatoi H.N., Das N.N., Pandey B.D. (2013). Chemical and microbial remediation of hexavalent chromium from contaminated soil and mining/metallurgical solid waste: A review. J. Hazard. Mater..

[22] Fu L., Feng A., Xiao J., Wu Q., Ye Q., Peng S. (2021). Remediation of Soil Contaminated with High Levels of Hexavalent Chromium by Combined Chemical-Microbial Reduction and Stabilization. J. Hazard. Mater..

[23] Ke Q., Ren J., Feng K., Zhang Z., Huang W., Xu X., Zhao L., Qiu H., Cao X. (2024). Crucial Roles of Soil Inherent Fe-Bearing Minerals in Enhanced Cr (VI) Reduction by Biochar: The Electronegativity Neutralization and Electron Transfer Mediation. Environ. Pollut..

[24] Song M., Zhou X., Wu J., Yang J., Wang X. (2026). Biochar from Wet-Process Phosphoric Acid in-Situ Modified Bagasse for Cr (VI) Removal. J. Clean. Prod..

[25] Hou B., Yang X., Wang L., Shi T., Akram M., Wang L., Wan J., Gao B., Pan J. (2024). Fe Doping Enhanced Cr (VI) Adsorption Efficiency of Cerium-Based Adsorbents: Adsorption Behaviors and Inner Removal Mechanisms. J. Colloid. Interface Sci..

[26] Simonin J.-P. (2016). On the Comparison of Pseudo-First Order and Pseudo-Second Order Rate Laws in the Modeling of Adsorption Kinetics. Chem. Eng. J..

[27] Chen M., He F., Hu D., Bao C., Huang Q. (2020). Broadened Operating pH Range for Adsorption/Reduction of Aqueous Cr (VI) Using Biochar from Directly Treated Jute (*Corchorus capsularis* L.) Fibers by H_3_PO_4_. Chem. Eng. J..

[28] Vitek R., Masini J.C. (2023). Nonlinear Regression for Treating Adsorption Isotherm Data to Characterize New Sorbents: Advantages over Linearization Demonstrated with Simulated and Experimental Data. Heliyon.

[29] Fei Y.-H., Li M., Ye Z., Guan J., Huang Z., Xiao T., Zhang P. (2022). The pH-Sensitive Sorption Governed Reduction of Cr(VI) by Sludge Derived Biochar and the Accelerating Effect of Organic Acids. J. Hazard. Mater..

[30] Lian G., Wang B., Lee X., Li L., Liu T., Lyu W. (2019). Enhanced Removal of Hexavalent Chromium by Engineered Biochar Composite Fabricated from Phosphogypsum and Distillers Grains. Sci. Total Environ..

[31] Zhu S., Huang X., Yang X., Peng P., Li Z., Jin C. (2020). Enhanced Transformation of Cr (VI) by Heterocyclic-N within Nitrogen-Doped Biochar: Impact of Surface Modulatory Persistent Free Radicals (PFRs). Environ. Sci. Technol..

[32] He M., Xu Z., Sun Y., Chan P.S., Lui I., Tsang D.C.W. (2021). Critical Impacts of Pyrolysis Conditions and Activation Methods on Application-Oriented Production of Wood Waste-Derived Biochar. Bioresour. Technol..

[33] Sireesha S., Sreedhar I. (2023). Holistic and Parametric Optimization Study on Cr (VI) Removal Using Acid-Treated Coco Peat Biochar Adsorbent. Bioresour. Technol. Rep..

[34] Sathasivam J., Rajaraman P.V., Narayanasamy S. (2025). Assessment of Cerium Adsorption Potential of Phosphoric Acid Activated Biochar in Aqueous System: Modelling and Mechanistic Insights. Environ. Res..

[35] Tan Y., Wang J., Zhan L., Yang H., Gong Y. (2024). Removal of Cr (VI) from Aqueous Solution Using Ball Mill Modified Biochar: Multivariate Modeling, Optimization and Experimental Study. Sci. Rep..

[36] Han L., Li J., Zhang T., Qu C., Yu T., Yang B., Shao Z. (2022). Removal of Cr (VI) by Biochar Derived via Co-Pyrolysis of Oily Sludge and Corn Stalks. Sci. Rep..

[37] Chen X., Dai Y., Fan J., Xu X., Cao X. (2021). Application of Iron-Biochar Composite in Topsoil for Simultaneous Remediation of Chromium-Contaminated Soil and Groundwater: Immobilization Mechanism and Long-Term Stability. J. Hazard. Mater..

[38] Nie X., Li G., Wang Y., Luo Y., Song L., Yang S., Wan Q. (2022). Highly Efficient Removal of Cr (VI) by Hexapod-like Pyrite Nanosheet Clusters. J. Hazard. Mater..

